# Operando Decoding of Surface Chemical and Thermal Events in Photoelectrocatalysis via a Lab‐Around‐Microfiber Sensor

**DOI:** 10.1002/advs.202310264

**Published:** 2024-04-30

**Authors:** Yunyun Huang, Caini Mou, Jiaxuan Liang, Jiaxin Wan, Pengwei Chen, Bai‐Ou Guan

**Affiliations:** ^1^ Guangdong Provincial Key Laboratory of Optical Fiber Sensing and Communications Institute of Photonics Technology Jinan University Guangzhou 511443 China; ^2^ College of Physics & Optoelectronic Engineering Jinan University Guangzhou 510632 China

**Keywords:** heat production, lab‐around‐microfiber sensor, operando decoding, photoelectrocatalysts, surface chemistry

## Abstract

Operando decoding of the key parameters of photo‐electric catalysis provides reliable information for catalytic effect evaluation and catalytic mechanism exploration. However, to capture the details of surface‐localized and rapid chemical and thermal events at the nanoscale in real‐time is highly challenging. A promising approach based on a lab‐around‐microfiber sensor capable of simulating photo‐electric catalytic reactions on the surface of optical fibers as well as monitoring reactant concentration changes and catalytic heat generation processes is demonstrated. Due to the penetration depth of submicron size and the fast response ability of the evanescent field, the lab‐around‐microfiber sensor overcame the difficulty of reading instantaneous surface parameters in the submicron range. This sensor operando dismantled the changes in reactant concentration and temperature on the catalyst surface induced by light and voltage, respectively. It also decoded the impact of catalyst composition on the adsorption efficiency and catalytic efficiency across various wavelengths and determined the synchronized occurrence of pollutant degradation and catalytic thermal effects. Stable correlations between the real‐time parameters and catalytic activities are obtained, helping to provide a basic understanding of the catalytic process and mechanism. This approach fills an important gap in the current monitoring methods of catalytic processes and heat production.

## Graphical abstract

To capture the details of surface‐localized and rapid chemical and thermal events at the microscale in real‐time, a promising approach based on a lab‐around‐microfiber sensor capable of simulating photocatalytic reactions on the surface of optical fiber and monitoring reactant concentration changes and catalytic heat generation processes is developed. It fills an important gap in current catalytic process monitoring methods.

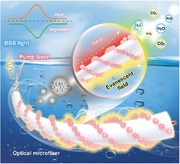

## 1 Introduction

The full potential of solar energy, which is sustainable, clean, safe, and pollution‐free, can be harnessed as chemical energy. This approach provides a viable and promising solution to the challenges posed by environmental pollution and energy scarcity.^[^ ^1^, ^2^, ^3^ ^]^ Photoelectrocatalytic technology is a promising approach for converting solar energy into chemical energy under mild conditions without causing any secondary pollution. It serves as a significant avenue to address environmental and energy challenges. The design and synthesis of photoelectrocatalysts have exhibited remarkable advancements on an annual basis.^[^ ^4^, ^5^ ^]^ In the catalytic process, the alteration of the surface reactant concentration and the generation of heat are pivotal parameters that characterize the catalytic efficacy and interpret the reaction mechanism.^[^ ^6^, ^7^ ^]^ These parameters subsequently govern the macroscopic reaction of the catalyst and exert a significant influence on the assessment of the structure‐activity correlation within the catalyst.^[^ ^8^ ^]^ To understand the photoelectrocatalysis mechanism and further improve its performance, monitoring and analyzing the local reactants and temperature changes at the catalyst surface from the macroscopic scale down to the microscopic scale are very important and challenging.^[^ ^9^ ^]^ Considerable studies have been performed to find new characterization techniques to provide valuable information regarding catalytic decomposition; these techniques include gas chromatography (GC)‐mass spectrometry,^[^ ^10^ ^]^ UV‒vis absorption spectroscopy,^[^ ^11^ ^]^ and Raman spectroscopy.^[^ ^12^ ^]^ Additionally, thermocouples,^[^ ^13^ ^]^ scanning thermal microscopy,^[^ ^14^ ^]^ and infrared thermal imagers^[^ ^15^ ^]^ have been used to monitor the temperature of catalysts. However, these methods usually require large and expensive instruments and complex operations.^[^ ^16^ ^]^ Moreover, their primary focus predominantly lies on macroscopic scales in a postmortem manner, thereby lacking the capability for continuous in situ monitoring.

At the microscopic level, particularly at the liquid‒solid interface, continuous on‐site monitoring of the reactant concentration and temperature on the catalyst surface poses a significant challenge. The challenge lies not only in the instrument's resolution but also in the rapid diffusion of matter and heat from the catalyst surface into the surrounding solution.^[^ ^17^ ^]^ The acquisition of transient surface localized material and thermal signals with high precision, rapidity, and high spatial resolution poses a formidable difficulty. In addition, fluctuations in the environmental substances and temperature inevitably interfere with the monitoring of the catalyst surface.^[^ ^18^, ^19^ ^]^ These problems have caused great obstacles in the study of catalytic mechanisms. To address this challenge, new sensor technologies capable of capturing crucial catalyst surface parameters at the submicron scale need to be developed.

Optical fiber sensors provide a promising approach to solving these challenges. They are made with chemically resistant silica glass and have high sensitivity, instantaneous response, and electrical immunity.^[^ ^20^, ^21^ ^]^ With an evanescent field at the submicron scale, optical fiber sensors interact with matter on their surface within the submicron scale.^[^ ^22^, ^23^ ^]^ The emerging “lab around fiber” provides many possibilities with functional nanostructures integrated into the external curved surface of the fiber.^[^ ^24^ ^]^ This configuration benefits from large interaction lengths and easier access to micro/nano technologies, facilitating the creation of multifunctional photonic elements along the fiber. Among the existing optical fiber sensors, optical microfibers, with compact sensing structures^[^ ^25^ ^]^ and the light of cladding modes penetrating the fiber environment, facilitate strong interactions between light and matter at submicroscopic scales. Consequently, they are highly suitable for conducting microscale experiments on photophysical and photochemical interactions.^[^ ^26^ ^]^

**Figure 1:**
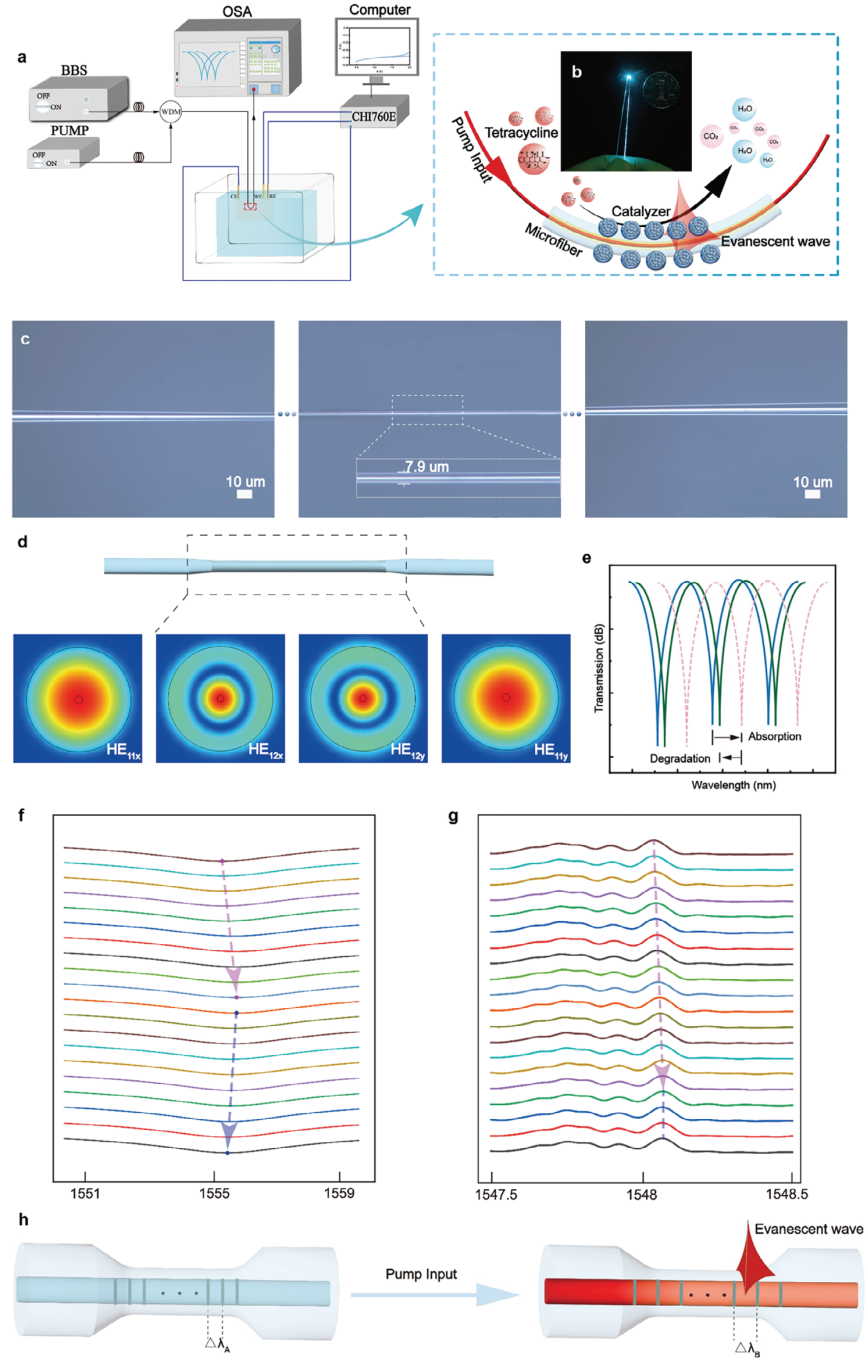
Schematic diagram of the setup of the lab‐around‐microfiber sensor. a) Schematic diagram of the lab‐around‐microfiber sensor. b) Photograph of the optical microfiber with catalysts on the surface. c) Optical microscopy photographs of the microfiber. The regions without evident changes between the transition region and waist region are not presented here due to space limitations. d) Z‐direction Poynting vector of the silica microfiber at 1550 nm with a diameter of 7.9 µm calculated by a numerical mode simulation software (HE_11_ and HE_12_ modes). e) Schematic illustration of the wavelength shifts in the transmission spectrum when an adsorption‐degradation process is sensed. The measured spectra in the photoelectroncatalytic process were recorded by f) the lab around microfiber and g) the lab around microfiber Bragg grating (microFBG). The spectra are presented as stacked images in which the adsorption (redshift) – degradation (blueshift) and heat production process (redshift) can be distinguished clearly. h) Schematic diagram of the temperature‐sensitive mechanism of the microFBG.

Herein, to address the challenges faced by the key parameter measurement of photocatalytic surfaces, a lab‐around‐microfiber sensor was proposed and developed for simulating photocatalytic reactions on the surface of optical fibers and operando decoding, as shown in **Figure** **1**. A single layer of photoelectrocatalysts was assembled on the microfiber surface in an orderly and quantitative manner, forming the lab‐around‐microfiber sensor (Figure 1a). The strong evanescent field interacted with the catalyst surface, capturing information on the surface reactant concentration and temperature changes. Due to the penetration depth of the submicron size and the fast response ability of the evanescent field, the lab‐around‐microfiber sensor overcame the difficulty of reading surface parameters in the submicron range. The sensor quickly recorded the transient reactant concentration and temperature signals on the catalyst surface. Using the bismuth oxide photoelectrocatalyst as an example, the lab‐around‐microfiber sensor operando decoded the changes in the reactant concentration and temperature on the catalyst surface induced by light and voltage, respectively. Stable and reproducible correlations between the real‐time parameters and catalytic activities were obtained, which helped to provide a basic understanding of the catalytic process and mechanism. This approach addressed an important deficiency in the current catalytic process and heat monitoring methods. It also provided a new method for the operando decoding of physical and chemical events at the submicron scale.

## 2 Results and Discussion

### 2.1 Setup and Sensing Mechanism of the Lab‐Around‐Microfiber Sensor

The morphology of the lab‐around‐microfiber sensor was tiny and flexible, as shown in Figure 1b. Its use could potentially enable the shift from the traditional testing scheme toward portable applications.^[^ ^27^ ^]^ The silica optical microfiber was tapered from a single‐mode fiber via a flame scanning method (Figure S1, Supporting Information).^[^ ^28^ ^]^ A 7.9 µm diameter, 0.9 mm length uniform region, and a transition region of 5 mm in length were fabricated on the taper (Figure 1c; Figure S2, Supporting Information). Biconically connected to single‐mode silica fibers that coupled the incoming probing light and collected the outgoing signal, the taper structure generated a fundamental mode (HE_11_ mode) and a higher‐order mode (HE_12_ mode).^[^ ^29^ ^]^ The HE_11_ mode propagated in the core of the silica microfiber (Figure 1d), while the HE_12_ mode spread out from the microfiber cladding to interact with the surface matter, i.e., the lab around microfiber. The interference between these two modes produced a fringe pattern (Figure 1e). The surface refractive index changes of the lab‐around‐microfiber sensor modulated the interferometric pattern shifts, as was determined by the following:^[^ ^25^ ^]^

(1) $$\frac{d\lambda }{dn_{ext}}=\lambda \times \frac{1}{\Gamma }\times \left(\frac{1}{\Delta n_{eff}}\times \frac{\partial ▵n_{eff}}{\partial n_{ext}}\right)$$

where $\Gamma \;=\;1-\frac{\lambda }{▵n_{eff}}\cdot \frac{d▵n_{eff}}{\partial n_{ext}}$, *n_ext_* is the surface refractive index of the microfiber, and △*n_eff_* is the effective refractive index difference between the HE_11_ and HE_12_ modes. Consequently, an increase in the surface refractive index of the microfiber resulted in a redshift of the wavelength, while a decrease in the surface refractive index led to a blueshift of the wavelength.

When the microfiber with a single layer of photocatalyst was immersed in the pollutant solution, the pollutants were adsorbed onto the surface of the photocatalyst, thereby forming the lab around microfiber in conjunction with the catalyst. Here, tetracycline was selected as the pollutant model since it is currently one of the most consumed antibiotics for human therapy, veterinary purposes, and agricultural activities.^[^ ^30^, ^31^, ^32^ ^]^ Thus, a submicron‐scale photoelectrocatalytic lab around microfiber was constructed. The size of this “lab” precisely matched the evanescent field penetration depth of the optical microfiber, thereby facilitating the optical microfiber's ability to perceive key parameters of the photoelectrocatalyst surface (Figure 1f). In addition, a fiber Bragg grating (FBG) was used to monitor the thermal effect on the catalyst surface (Figure 1g). By monitoring the wavelength shift of the Bragg core mode in reflection, FBGs are widely used for temperature measurement.^[^ ^33^, ^34^ ^]^ Using the core mode, the corresponding wavelength shift was not affected by the surface refractive index change. Without additional strain, the shift in the resonance wavelength with temperature is expressed as Equation (2):^[^ ^35^ ^]^

(2) $$\Delta \lambda_{B}=\lambda_{B}\times K_{T}\times ▵T$$

where *λ_B_* is the resonance wavelength, △*T* is the change in temperature and the coefficient *K_T_* is the temperature sensitivity of the resonance wavelength shift (△*λ_B_*). Thus, the surface temperature rise induced by heat production in photoelectrocatalysis could be recorded by the FBG. As shown in Figure 1h, when the catalyst layer generated heat, there was an increase in △*λ_B_*, and a corresponding redshift was observed in the spectrum (Figure 1g). The successful construction of a lab‐around‐microfiber sensor enables the in situ and real‐time monitoring of catalytic reactions and catalytic heat generation.

### 2.2 Synthesis of Bi_2_O_3_‐Based Catalysts

**Figure 2:**
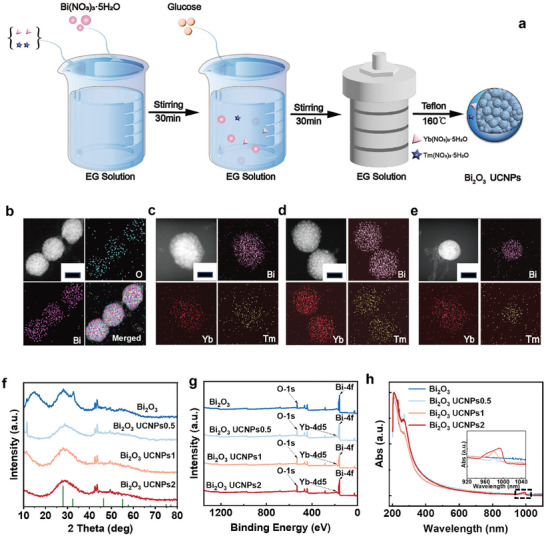
Design of the photoelectrocatalysts and preparation of the lab‐around‐microfiber sensor. a) Schematic drawing of the synthesis process of the Bi_2_O_3_ nanospheres and Bi_2_O_3_‐UCNPs. STEM image and corresponding element mapping of the b) Bi_2_O_3_ nanospheres, c) Bi_2_O_3_‐UCNPs with 0.05 g Yb^3+^ and 0.0075 g Tm^3+^ codoping (Bi_2_O_3_‐UCNPs0.5), d) Bi_2_O_3_‐UCNPs with 0.1 g Yb^3+^ and 0.015 g Tm^3+^ codoping (Bi_2_O_3_‐UCNPs1), e) Bi_2_O_3_‐UCNPs with 0.2 g Yb^3+^ and 0.03 g Tm^3+^ codoping (Bi_2_O_3_‐UCNPs2). The scale bar in b–e is 50 nm. f) XRD patterns, g) XPS survey spectra, and h) UV‒visible absorption spectra of the photoelectrocatalysts.

The catalyst model chosen for this study was Bi_2_O_3_, which serves as a representative UV/visible light catalyst.^[^ ^36^ ^]^ In order to address the issue of light absorption in the UV range by the catalyst and its low utilization rate of solar energy, a combination of upconversion nanocrystals and semiconductors can be employed. Within these materials, upconversion nanocrystals serve as a medium for converting near‐infrared (NIR) photons into UV photons to activate the semiconductor.^[^ ^36^ ^]^ In this work, a series of Bi_2_O_3_‐lanthanide ion‐doped nanospheres (Bi_2_O_3_‐UCNPs) was developed. The synthesis of Bi_2_O_3_‐based photoelectrocatalysts is illustrated in **Figure** **2**a. In the presence of glucose and ethylene glycol, Bi^3+^ was synthesized to form Bi_2_O_3_ under high temperature and pressure.^[^ ^37^ ^]^ The scanning transmission electron microscopy (STEM) images and their corresponding element maps were obtained by energy dispersive spectroscopy (EDS) in Figure 2b–e, and the transmission electron microscopy (TEM) images in Figure S3 (Supporting Information), showed the morphology of the Bi_2_O_3_ and the Bi_2_O_3_‐UCNPs. For Bi_2_O_3_, the densely packed agglomerate‐structured nanospheres with a uniform spherical morphology and a diameter of ≈100 nm were synthesized. Yb^3+^ and Tm^3+^‐doped bismuth hydroxide precursors were coated on the surface through a precipitation process (Figure 2a). Under hydrothermal treatment, uniform Yb^3+^/Tm^3+^‐doped Bi_2_O_3_‐UCNPs were obtained. The elemental mapping images showed that Yb and Tm were homogeneously distributed in Bi_2_O_3_‐UCNPs. X‐ray powder diffraction (XRD) patterns were used to characterize the crystal structure of the photocatalysts. Figure 2f presents the XRD patterns of Bi_2_O_3_ with various ratios of rare earth ion doping. The sample diffraction peaks were indexed to the rhombohedral phase of Bi [space group: *R3m* (166)] (JCPDS 44–1246).^[^ ^38^ ^]^ This result indicated that the Bi_2_O_3_ crystal structure formed in all photocatalysts and was not altered by the addition of Yb^3+^ and Tm^3+^ ions. To further clarify the chemical composition of the photocatalysts, X‐ray photoelectron spectroscopy (XPS) was performed, as shown in Figure 2g and Figure S4 (Supporting Information). The XPS survey indicated the presence of Bi and O in Bi_2_O_3_ as well as the presence of Bi, O, and Yb in Bi_2_O_3_‐UCNPs. The EDS results in Figures S5–S8 and Tables S1–S4 (Supporting Information) also confirmed the presence of Bi and O in Bi_2_O_3_ as well as the presence of Bi, O, Yb, and Tm in Bi_2_O_3_‐UCNPs. The Bi 4f spectra of the samples had two dominant peaks at 164.5 and 159.2 eV, confirming the Bi─O bonds of Bi_2_O_3_.^[^ ^37^ ^]^ These results were consistent with the EDS mapping results, further confirming the Bi_2_O_3_ structure and the rare earth ion doping in Bi_2_O_3_‐UCNPs. The UV‒visible absorption spectra in Figure 2h showed that Bi_2_O_3_ absorbed UV/visible light, while Bi_2_O_3_‐UCNPs absorbed near‐infrared light at 980 nm. According to the UV–visible absorption spectrum of Bi_2_O_3_, the bandgap of Bi_2_O_3_ could be calculated^[^ ^39^ ^]^ to be 2.64 eV as shown in Figure S9 (Supporting Information). The upconversion PL spectra of the catalysts under excitation at 980 nm wavelength are shown in Figure S10 (Supporting Information). No PL emission was observed in the Bi_2_O_3_ nanospheres, while for Bi_2_O_3_‐UCNPs, three upconversion peaks, namely one UV emission and two blue emissions, could be observed in the spectra. The instrument's limitations restricted the measurement of wavelengths to a minimum of 350 nm. According to the reported work,^[^ ^36^ ^]^ PL spectra have another peak at 346 nm, but our instrument cannot detect it. Hence, the rare earth nanocrystals exhibit absorption of 980 nm light and subsequent conversion into UV light to effectively activate the catalytic properties of Bi_2_O_3_.

### 2.3 Lab‐Around‐Microfiber Sensor Fabrication

**Figure 3:**
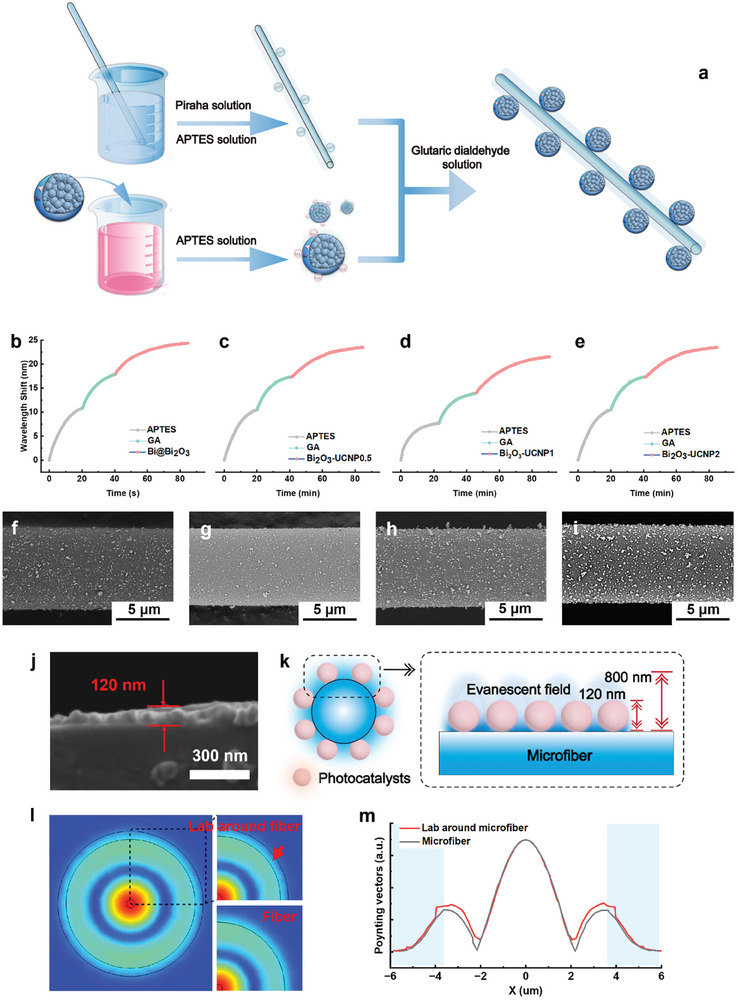
Lab around microfiber functionalization. a) Schematic of the immobilization of photo electrocatalysts on microfiber surfaces. Wavelength shifts in the transmission spectrum recorded in the functionalization process of the b) Bi_2_O_3_ nanospheres, c) Bi_2_O_3_‐UCNPs0.5, d) Bi_2_O_3_‐UCNPs1, and e) Bi_2_O_3_‐UCNPs2. SEM images of microfiber surfaces of the f) Bi_2_O_3_ nanospheres, g) Bi_2_O_3_‐UCNPs0.5, h) Bi_2_O_3_‐UCNPs1, and i) Bi_2_O_3_‐UCNPs2. j) SEM side‐view image of microfiber surface with Bi_2_O_3_ nanospheres. k) Schematic diagram of the photocatalysis inside the evanescent field of the microfiber. l) Z‐direction Poynting vector of the lab around microfiber calculated by numerical mode simulation software (HE_12_ mode). m) Transverse electric field amplitude distributions of the HE_12_ mode of the lab around microfiber and silica microfiber calculated by numerical mode simulation software.

As demonstrated in **Figure** **3**a, before fabricating the lab‐around‐microfiber sensor, the surfaces of Bi_2_O_3_ nanospheres and Bi_2_O_3_‐UCNPs were modified by amino groups via the reaction in a 3‐aminopropyl‐triethoxysilane (APTES) solution.^[^ ^40^ ^]^ The silica surface of the optical microfiber was sequentially modified using a piranha solution and APTES solution, resulting in a substantial increase in the density of the surface amino groups. After the respective functionalization, the photoelectrocatalysts were assembled on the surfaces of optical microfibers through the crosslinking of glutaraldehyde (GA) and the amino groups on both sides.^[^ ^41^ ^]^ Figure 2b–e and Figure S11 (Supporting Information) show the wavelength shifts recorded by the optical microfibers in the fabrication process of the lab‐around microfiber sensor. During the catalyst assembly process, the spectra gradually redshifted and reached equilibrium. The similar wavelength in the fabrication processes from Bi_2_O_3_‐around‐fiber to Bi_2_O_3_‐UCNPs‐around‐fiber sensors indicated a similar amount of assembly on microfiber surfaces. The morphologies of lab‐around‐microfiber sensors were investigated by scanning electron microscopy (SEM) images, as shown in Figure 2f–i. Single‐layer photocatalysts were immobilized on microfiber surfaces (Figure 2j). The thickness of this layer was ≈120 nm, which was within the evanescent field of the microfiber. The catalyst occupation of microfiber surface area was 11.806 ± 1.001% (Figures S12–S15 and Tables S5–S8, Supporting Information). Moreover, the same modification approach was used to fabricate catalyst layers on the surface of micro‐FBGs for monitoring catalytic thermal generation. The resulting catalyst occupation of the micro‐FBG surface area was 11.118 ± 0.809% (Figures S16–S19 and Tables S9–S12, Supporting Information). This result indicated that quantitative and ordered arrangements of monolayer catalysts on optical fiber surfaces were attained. When a catalyst layer with a thickness of ≈120 nm was assembled onto the microfiber surface, it was positioned within the evanescent field range of the microfiber (penetration depth of ≈800 nm, Figure 3k). The incorporation of a high refractive index catalyst layer onto the microfiber surface facilitated enhanced evanescent field concentration within the catalyst layer, thereby enabling more sensitive detection of material and thermal variations in the catalyst layer (Figure 3l,m). The successful fabrication of the lab‐around‐microfiber sensor paved the way for operando decoding of surface chemical and thermal events in photo‐electric catalysis.

### 2.4 UV/Visible Light‐Induced Photoelectrocatalysis Process

**Figure 4:**
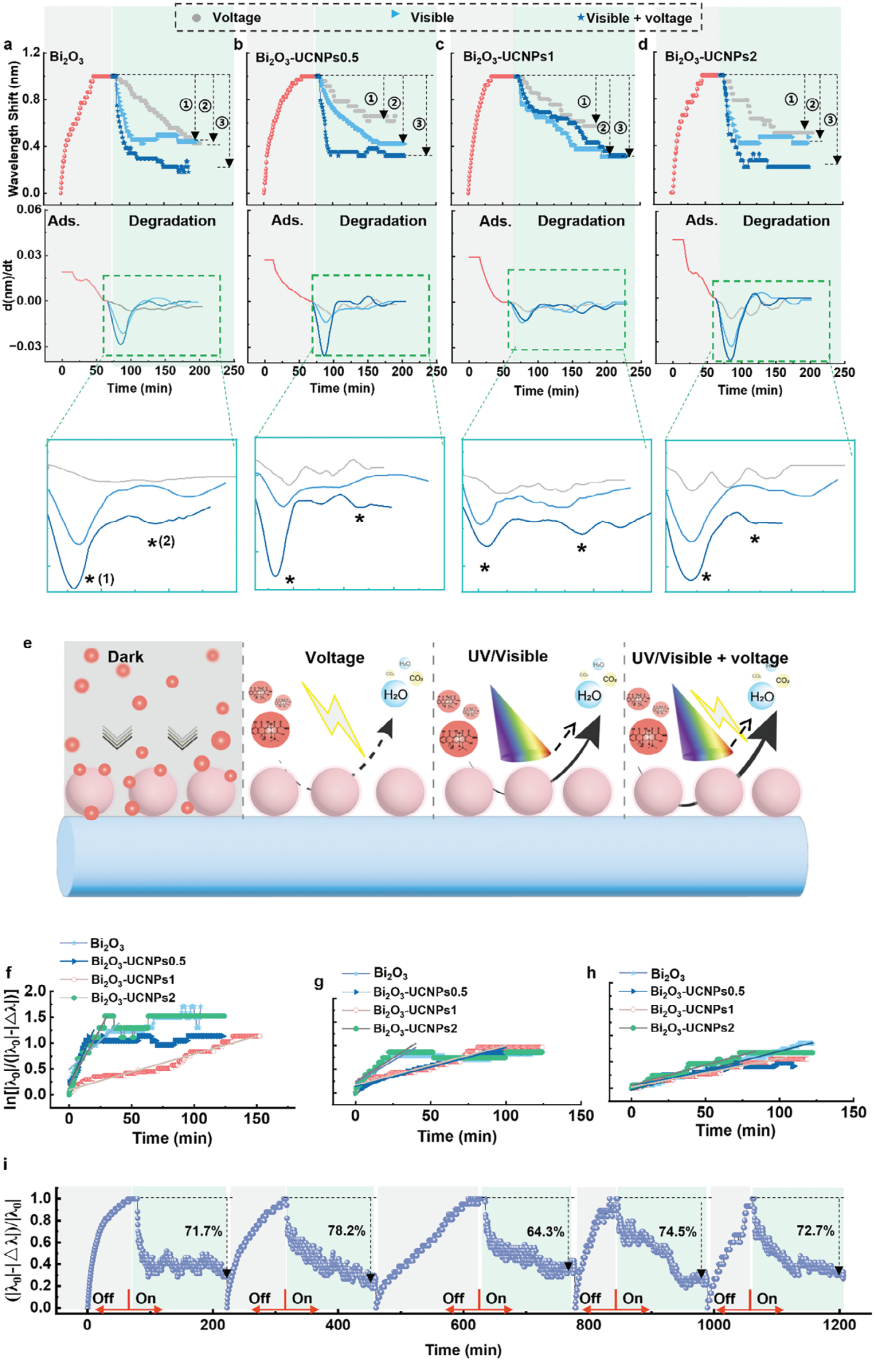
Photoelectrocatalytic process driven by UV/visible light monitored using lab‐around‐microfiber sensors. Wavelength shift recorded by the lab‐around‐microfiber sensors in the tetracycline adsorption and degradation process and the corresponding differential wavelength shifts over various catalysts: a) Bi_2_O_3_, b) Bi_2_O_3_‐UCNP0.5, c) Bi_2_O_3_‐UCNP1, and d) Bi_2_O_3_‐UCNP2. Degradation process: the photothermal effect caused by irradiation deduced by subtracting the baselines. The baselines: the wavelength shift obtained in water with various catalysts around microfibers. e) Schematic diagram of the photoelectrocatalysis process of tetracycline over catalysts on the optical microfiber. f–h) Kinetics of the photoelectrocatalysis process of tetracycline over catalysts on the optical microfiber. (mean ± SD, n = 3) (**f** UV/visible light‐voltage; UV/visible light; voltage.) i) Cycles of the adsorption and degradation of tetracycline over Bi_2_O_3_ (recorded by the lab‐around‐microfiber sensor).

The changes in reactant concentration on the catalyst surface of Bi_2_O_3_ and Bi_2_O_3_‐UCNPs in the photoelectrocatalysis process were assessed, as shown in **Figure** **4**. The optical stability of the lab‐around‐microfiber sensor in aqueous solution was evaluated, as presented in Figure S20 (Supporting Information). The spectrum was stable in an aqueous environment. The adsorption and degradation processes of the pollutants on the catalyst surface were recorded using the lab‐around‐microfiber sensor based on this stability. When the lab‐around‐microfiber sensor was exposed to the tetracycline solution in the dark, the tetracycline molecules were adsorbed onto the catalyst surfaces. This process resulted in redshifts in the transmission fringe in the first 50 min, and equilibrium was reached (Figure 4a–d; Figure S21, Supporting Information). The adsorption process significantly increased the reactant concentration on the surface of the lab‐around‐microfiber sensor, and a catalytic layer was formed that included the catalysts and pollutants around the microfiber within the evanescent field. Under UV/visible light irradiation and electrical stimulation, UV/visible light irradiation alone, and electrical stimulation alone, the optical microfibers with various catalysts recorded blueshifts in the transmission fringes (Figure S22, Supporting Information). Since the wavelength shift was not only modulated by the surface refractive index change (the matter change) around the microfiber but also was influenced by the surface temperature, the irradiation‐on‐microfiber‐induced thermal effect (the blueshift recorded without pollutant) was evaluated as the baseline and subtracted from the blueshifts recorded when the lab‐around‐fiber sensors were stimulated by UV/visible light + voltage, UV/visible light, and voltage (Figure S23, Supporting Information line ②‐line ①). The results are shown in the “Degradation step” in Figure 4a–d. Since the catalyst particles were immobilized onto the fiber surface using APTES, concerns regarding adhesion arose. To eliminate any potential influence of APTES on catalytic efficiency evaluation, UV–visible absorption spectroscopy was employed to assess the degradation efficiency of tetracycline on both APTES‐modified and unmodified catalysts (Figure S24, Supporting Information). The results demonstrated comparable pollutant degradation efficiencies for both types of catalysts, thereby excluding the impact of the organic linker on the catalytic evaluation outcomes.

The differentials of the wavelength shifts obtained by taking the derivative of the wavelength shift level with respect to time are further shown in the second row of Figure 4a–d to investigate the instantaneous adsorption/degradation rates. This represents the change rate of wavelength shifts.^[^ ^16^ ^]^ The results showed that in the adsorption step, the adsorption rates of pollutants on the surfaces of various catalysts exhibited a consistent pattern, characterized by an initial rapid phase followed by a gradual deceleration and ultimately approached equilibrium. This was due to the similarity of the morphologies of these catalysts^[^ ^42^ ^]^ as shown in Figure 4e. In the degradation step, pollutants still exhibited comparable degradation rates on various catalyst surfaces. Compared to the gradual and consistent changes observed in the spectrum under voltage, both UV/visible light irradiation and UV/visible light irradiation + voltage exhibited dual degradation rate peaks (a primary peak and a secondary peak). The above evidence substantiated the pivotal role of UV/visible light irradiation in instigating catalytic reactions mediated by these photoelectrocatalysts. Under UV/visible light irradiation, the accumulation of reactants produced the first degradation peak, while the consumption of products resulted in the second degradation peak^[^ ^43^ ^]^ as shown in Figure 4e. Therefore, the lab‐around‐microfiber sensor enabled the assessment of real‐time kinetics in photoelectrocatalytic reactions, facilitating inference of the correlation between catalyst structure and performance. Additionally, the sensor‐enabled separate assessment of light‐induction and voltage stimulation effects on catalytic reactions to determine the dominant excitation source.

Moreover, the relative wavelength shift (|△λ|/|λ_0_|) was used to evaluate the photodegradation performance, where Δ*λ* represents the real‐time blueshift of the wavelength recorded by the microfiber (excluding temperature effects), and *λ_0_* denotes the total redshift of the spectrum caused by tetracycline adsorbed on the catalyst surface on the microfiber. The degradation processes in Figure 4a–d recorded by the lab‐around‐microfiber sensors also demonstrated the degradation efficiencies of tetracycline on various catalyst surfaces, which are listed in Table 1. Under identical excitation sources, the degradation efficiency of tetracycline on catalysts doped with or without rare earth ions exhibited no significant disparity. Under UV/visible light irradiation, the degradation efficiency of tetracycline approached that achieved under the synergistic effect of UV/visible light and voltage stimulation, indicating that UV/visible light irradiation played a dominant role with voltage stimulation acting as a supplementary factor. This result demonstrated the efficacy of the lab‐around‐microfiber method in decomposing and assessing the impact of multiple excitation sources on the catalytic efficiency of photoelectrocatalysts.

**Table 1:** Degradation efficiencies of tetracycline on various catalyst surfaces.

	Bi_2_O_3_	Bi_2_O_3_‐UCNPs0.5	Bi_2_O_3_‐UCNPs1	Bi_2_O_3_‐UCNPs2
UV/visible light + voltage	③77.328%	③67.791%	③67.707%	③78.156%
UV/visible light	①55.833%	②57.688%	②61.953%	②57.869%
Voltage	②57.15%	①38.287%	①42.337%	①48.976%
NIR light + voltage	②45.842%	③58.344%	②81.817%	②87.05%
NIR light	①0%	①23.999%	①51.357%	①69.198%

Since the pollutant removal behavior over various catalysts follows the pseudo‐first‐order kinetics rate law^[^ ^44^ ^]^ as shown in Equation (3), the first‐order kinetic rate constant *k* was evaluated to further assess the catalytic performance of the catalysts.

(3) $$ln\left(C_{0}/C\right)=kt$$

where *C*_0_ is the tetracycline concentration on the catalyst surface after the initial adsorption‐desorption, *C* is the concentration at different irradiation times, and *t* is the irradiation time. The first‐order kinetic rate constant *k* can be expressed as the proportionality between wavelength shifts and pollutant concentrations on the microfiber surface:

(4) $$k=\left[ln\left|\lambda_{0}\right|/\left(\left|\lambda_{0}\right|-\left|\Delta \lambda \right|\right)\right]/t$$

The ln |*λ_0_* |/(|*λ_0_* |‐|*△λ*|) values over irradiation time *t* were calculated as shown in Figure 4f–h. The resulting *k* values are listed in Table 2. The detection results demonstrated that the degradation rate of pollutants on the surface of various catalysts was significantly enhanced under UV/visible light irradiation compared to that without light irradiation. Under the same excitation source (UV/visible light irradiation, electrical stimulation, or UV/visible light irradiation + electrical stimulation), no significant difference in the degradation rate of pollutants on various catalyst surfaces was observed. Thus, different catalysts possessed similar absorption capacities for UV/visible light. The prepared lab‐around‐microfiber sensor demonstrated its capability to assess the excitation ability of diverse excitation sources on catalytic reactions by evaluating the degradation rate of pollutants on the catalyst surface, thereby facilitating a comprehensive analysis of the correlation between catalyst structure and catalytic efficacy. Furthermore, the lab‐around‐microfiber sensor underwent at least five adsorption‐degradation cycles on the catalysts (Bi_2_O_3_ in Figure 4i). This result indicates that the catalysts can be used repeatedly for pollutant degradation. Therefore, the lab‐on‐microfiber sensor can also assess the catalyst's reproducibility.

**Table 2:** *k* values of the various catalyst surfaces.

	Bi_2_O_3_[min^−1^]	Bi_2_O_3_‐UCNPs0.5[min^−1^]	Bi_2_O_3_‐UCNPs1[min^−1^]	Bi_2_O_3_‐UCNPs2 [min^−1^]
UV/visible light + voltage	0.02247	0.05444	0.00691	0.04374
UV/visible light	0.0186	0.00821	0.00838	0.02025
Voltage	0.00729	0.00556	0.00541	0.00772
NIR light + voltage	0.0045	0.00327	0.00992	0.01463
NIR ligh	0.0000919204	0.00163	0.00574	0.00987

### 2.5 NIR Light‐Induced Photoelectrocatalysis Process

**Figure 5:**
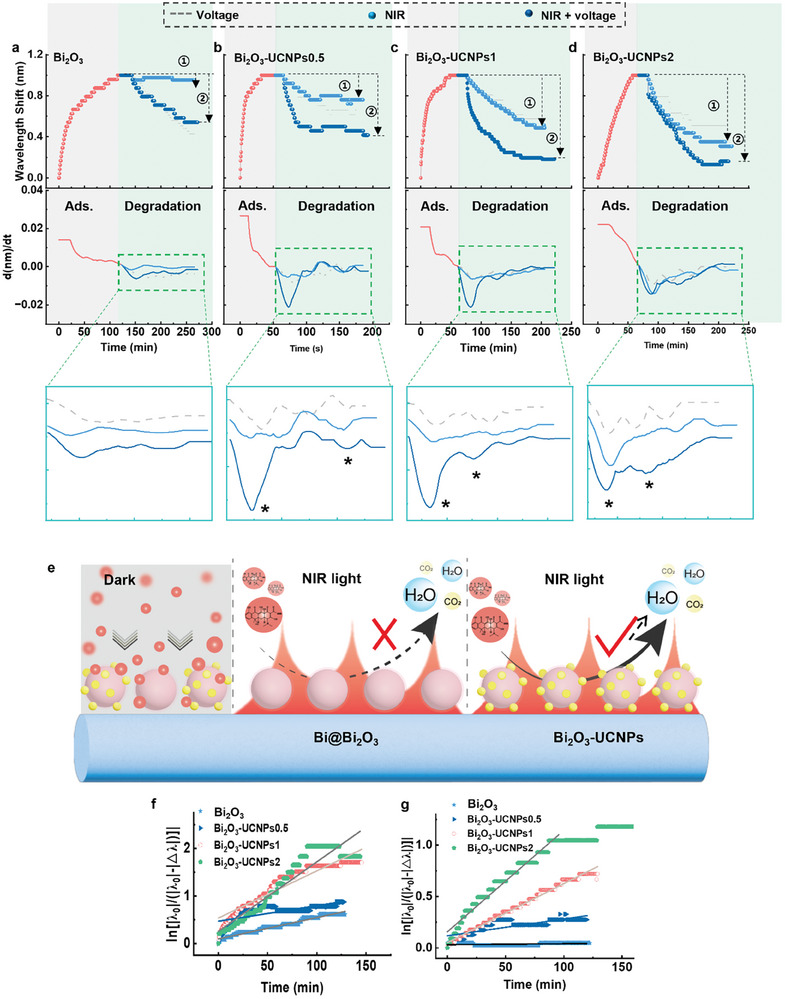
Photoelectrocatalytic process driven by NIR light monitored using lab‐around‐microfiber sensors. Wavelength shift recorded by the lab‐around‐microfiber sensors in the tetracycline adsorption and degradation process and the corresponding differential wavelength shifts over various catalysts: a) Bi_2_O_3_, b) Bi_2_O_3_‐UCNP0.5, c) Bi_2_O_3_‐UCNP1, and d) Bi_2_O_3_‐UCNP2. The degradation process: the photothermal effect caused by irradiation deduced by subtracting the baselines. The baselines: the wavelength shift obtained in water with various catalysts around microfibers. e) Schematic diagram of the photoelectrocatalysis process of tetracycline over Bi_2_O_3_ and Bi_2_O_3_‐UCNPs on the optical microfiber under NIR light. f,g) Photocatalytic degradation kinetics of tetracycline over various catalysts on the optical microfiber under NIR light + voltage or only NIR light. (mean ± SD, n = 3) (NIR light + voltage; NIR light.).

The changes in reactant concentration on the catalyst surface of Bi_2_O_3_ and Bi_2_O_3_‐UCNPs in the photoelectrocatalysis process induced by NIR light were evaluated, as shown in **Figure** **5**. The adsorption of pollutants also led to redshifts in the spectra. The observed redshift processes shown in Figures 5a–5d exhibited similarities, further indicating the resemblance in catalyst morphologies. In the stage of pollutant degradation, the microfiber recorded a significant difference between ion‐doped and non‐ion‐doped catalysts. After accounting for the baseline induced by photothermal effects (Figures S25, Supporting Information), discernible variations in wavelength shifts were observed among the catalysts during the process of pollutant degradation. Under the combined effect of NIR light irradiation and voltage, as well as individual treatments with laser irradiation or voltage, the degradation rate of pollutants on the catalyst surface without ion doping exhibited a sluggish trend (Figure 5a). This result demonstrated a disparity in the catalytic performance between Bi_2_O_3_ under UV/visible light irradiation and NIR light, highlighting the differential impact of excitations from these two wavelengths on catalyst efficiency. Conversely, the degradation rates of pollutants on the surface of ion‐doped catalysts were significantly enhanced when subjected to NIR light irradiation combined with electrical stimulation compared to either NIR light irradiation alone or voltage alone. Furthermore, an increase in doping concentration led to a convergence between the degradation rates observed under NIR light irradiation alone and those observed under NIR light irradiation combined with voltage. This observation indicated that Bi_2_O_3_ did not exhibit any absorption effect on NIR light, and the catalyst's response to NIR light was attributed to the upconversion of rare earth ions.^[^ ^45^ ^]^ As the doping concentration increased, the catalyst exhibited a stronger absorption effect on NIR light, leading to an enhanced catalytic response (Figure 5e). Therefore, the spectral data acquired from the lab‐around‐microfiber sensor could be utilized to infer the correlation between catalyst structure and catalytic performance. In addition, the relative wavelength shift (|△λ|/|λ_0_|) and *k* values were used to evaluate the photodegradation performance under NIR light irradiation combined with electrical stimulation and under NIR light irradiation alone, as shown in the degradation stage in Figure 5a–d and Figure 5f,g, and the results are provided in Tables 1 and 2. Under NIR irradiation, the degradation rate of pollutants on the surface of Bi_2_O_3_ was negligible, indicating its low absorption capacity for NIR light (Figure 5e), which aligned with the findings from our absorption spectrum analysis. With the increase in the doping concentration of rare earth ions, the degradation rate of pollutants on the catalyst gradually increased under NIR light irradiation, even surpassing the degradation rate observed under UV/visible light irradiation. Consequently, employing a lab‐around‐microfiber sensor further substantiated the absorption efficacy of rare earth ion‐doped catalysts toward NIR light, aligning consistently with their absorption spectra.

### 2.6 Temperature Change Monitoring

**Figure 6:**
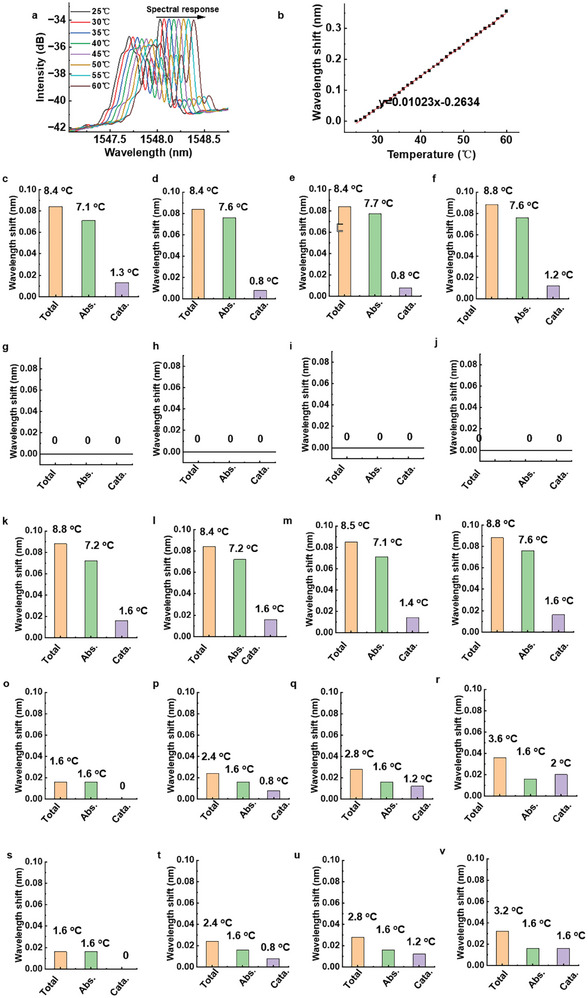
Temperature change monitoring. a) Measurement spectra and b) wavelength shifts of the microFBG with temperature increasing. c–v) Wavelength shifts of the microFBG and the corresponding temperature rises in the photoelectrocatalysis process. (mean ± SD, n = 3) Under UV/visible light, under voltage, under UV/visible light + voltage, **o‐r** under NIR light, under NIR light + voltage; Bi_2_O_3_, Bi_2_O_3_‐UCNP0.5, Bi_2_O_3_‐UCNP1, and Bi_2_O_3_‐UCNP2.).

In addition to the variation in reactant concentrations, the temperature fluctuations at the catalytic interface are a crucial parameter influencing the catalytic process. It is reported that in photoelectrocatalysis, the inevitable generation of heat in turn drives the catalysts' microscopic reactions and influences the quantitative structure‐activity relationships of the catalyst.^[^ ^46^ ^]^ Hence, real‐time and in situ monitoring of catalytic heat production is an essential aspect for tracking key parameters during the catalytic process. Here, an FBG was written in the microfiber core to monitor the temperature changes (heat production) of the photoelectrocatalytic process. The temperature sensitivity results of the micro‐FBG are illustrated in **Figure** **6**a,b; a linear correlation was observed between the wavelength shift and temperature increase. The heat‐measurement results of various catalysts under various excitations are shown in Figure 6c–v and Figure S26 (Supporting Information). According to the results in Figure S26 (Supporting Information), during the photo‐involved catalytic process, the lab‐around‐microFBG sensors recorded wavelength shifts ranging from 0.04 to 0.1 nm (red lines in Figure S26, Supporting Information), corresponding to temperature rises of 4 to 10 °C. These heat rises comprised both the photoheat generated by light absorption and the heat generated by the catalytic reaction. We need to separate these two types of heats. In this study, the wavelength shifts were detected when the the catalysts were under the simulations without pollutants to strip out the thermal effects of the catalysts on the light absorption (black lines in Figure S26, Supporting Information). It reveals that the wavelength shifts induced by this part of heat generation of various catalysts in this process were in the range of 0.015–0.07 nm, corresponding to a temperature elevation of 1.5–7 °C (indicated as Abs. Column in Figure 6c–v). The spectral results indicate a significantly higher heat generation in the catalyst absorption of UV/visible light compared to that of NIR light, which can be attributed to the lower power and more focused nature of NIR light, resulting in reduced heat loss.

Furthermore, we subtracted the wavelength shifts recorded by the microFBG caused solely by the photothermal effect from the overall wavelength shifts recorded by the microFBG caused by total heat generation (red lines in Figure S26, Supporting Information). Thus, the changes in heat and the resulting temperature increases generated by the catalytic process are illustrated in Figure 6c–v (indicated as Cata. column) and Figure S27 (Supporting Information). During the initial 50 min of the catalytic reaction, a gradual increase in catalytic heat generation was observed until the reaction reached equilibrium, mirroring the degradation process of the pollutants. This part of heat resulted from the catalytic reaction, thereby establishing a direct correlation with the degree of catalysis. Whether the catalysts were undoped or doped, no significant difference was observed in their catalytic thermal effect under UV/visible light irradiation. The temperature increase induced by reactions was ≈1.5 °C during these processes (Figure 6c–f). This finding was further supported by the wavelength shifts of the pollutant degradation process recorded using the lab‐around‐microfiber sensors. In contrast, when exposed to NIR light, the temperature increase induced by reactions of undoped and doped catalysts exhibited noticeable differences. An increase in the amount of rare earth doping enhanced the heat production effect of the catalytic process (Figure 6o–r). This result was also consistent with the degradation ability curves of pollutants on different catalyst surfaces under NIR light, confirming that the difference in the degradation ability of pollutants on various catalyst surfaces originated from the difference in the absorption ability of catalysts with different ion doping ratios to NIR light. The generation of heat was positively associated with this phenomenon. Notably, the thermal impact resulting from the voltage stimulation in the photoelectrocatalytic process was virtually unnoticeable; thus, the heat generation within this catalytic system was insignificant (Figure 6g–j). Therefore, the heat generation of catalytic reaction remained consistent with that of light irradiation alone when stimulated by UV/visible light +voltage and NIR+ voltage (Figure 6k–n,s–v). Furthermore, Figure S28 (Supporting Information) indicates the reliability of this lab‐around‐microfiber sensor toward temperature monitoring in degradation cycles.

**Figure 7:**
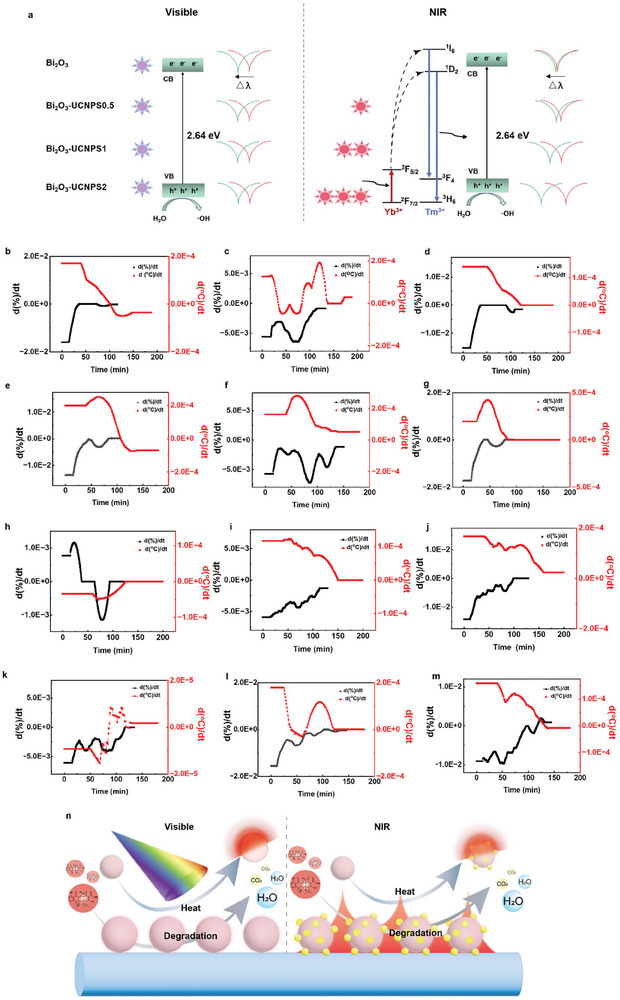
Consistency of heating and degradation. a) Schematic illumination of the upconversion and photocatalysis mechanism of the Bi_2_O_3_‐UCNPs under visible light and NIR light irradiations. b–m) relationship between spectral derivatives of the pollutant degradation process ((|λ_0_|‐|△λ|)/|λ_0_| over time) and derivatives of the temperature changes. Under UV/visible light, under UV/visible light + voltage, under NIR light, under NIR light + voltage; Bi_2_O_3_, Bi_2_O_3_‐UCNP1, and Bi_2_O_3_‐UCNP2.) n) Schematic diagram of the synchronous process of pollutant degradation and heat production.

The roles of illumination and voltage in photoelectrocatalysis, as well as the influence of different wavelengths of light on the catalytic effect, have been extensively investigated and discussed. The concentration of the reactants and heat changes on the catalyst surface during the catalytic process are crucial factors for investigating the catalytic effect. Therefore, our lab‐around‐microfiber sensor, which could achieve in situ and operando optical detection of the concentration of the reactants and heat production on the catalyst surface, was of great significance. The optical response could elucidate the distinct influences of illumination and voltage on the catalytic process, effectively indicating the auxiliary excitation effect of voltage.^[^ ^47^ ^]^ This response enabled an in‐depth analysis of how the different wavelengths of illumination impacted the catalytic efficiency, thereby facilitating a comprehensive understanding of the correlation between the catalyst's internal structure and its performance. Specifically, according to the absorption spectra presented in Figure 2h, Bi_3_O_2_ exclusively absorbs UV/visible light, while Bi_2_O_3_‐UCNPs exhibits absorption of both UV/visible and NIR light at ≈980 nm. Under UV/visible light irradiation, both doped and undoped catalysts exhibited similar light absorption capacities, leading to comparable catalytic effects. As demonstrated in **Figure** **7**a, in this process, UV/visible light‐triggered photogenerated electrons in the valence band of bismuth trioxide to the conduction band, resulting in the formation of a photoinduced electron‐hole (e^−^‐h^+^) pair.^[^ ^48^ ^]^ The bandgap of Bi_3_O_2_ was 2.64 eV and the flat‐band potentials (E_fb_) were determined to be to be −0.433 V (Figure S29, Supporting Information). The E_VB_ could be calculated to be 2.183. Thus, both doped and undoped catalysts exhibited similar light absorption capacities, leading to comparable catalytic effects; this was manifested in the spectra through similar wavelength shifts (degradation efficiency) and rates of wavelength shift (degradation rate). Under the illumination of NIR light, the Yb^3+^, Tm^3+^‐dopants converted NIR light to visible emission to trigger the photocatalysis reaction catalyzed by bismuth trioxide (according to the result in Figure S10, Supporting Information), effectively breaking H_2_O and forming hydroxyl free radicals (·OH) to induce the degradation of pollutants.^[^ ^42^ ^]^ In this process, a higher dopant concentration correlated to a greater ability for the absorption and conversion of NIR light into visible light. Consequently, more e^−^‐h^+^ pairs were excited in the catalysts, leading to enhanced catalytic efficiency and degradation rate; this was manifested in the spectra through larger wavelength shifts (degradation efficiency) and rates of wavelength shift (degradation rate).

Additionally, our lab‐around‐microfiber sensor enabled the assessment of the correlation between the catalysis process (the degradation of pollutants) and the generation of catalytic thermal effects. The spectral derivatives of the pollutant degradation process ((|λ_0_|‐|△λ|)/|λ_0_| over time), depicted as black lines in Figure 7b–m and Figure S30 (Supporting Information), were used to represent the instantaneous degradation rate of pollutants. Similarly, the derivatives of the temperature changes induced by the catalytic thermal effect were utilized to show the instantaneous change rate of the catalytic temperature rise (red lines in Figure 7b–m; Figure S30, Supporting Information). Based on the consistent relationship between the degradation rate of pollutants and the temperature rise rate, the catalytic process was accompanied by a photoinduced catalytic heat effect, highlighting the significance of the thermal effect in pollutant removal. The heat generation and pollutant degradation reactions were two simultaneous processes and primarily arose as the reactions responding to the absorption light (Figure 7n). Furthermore, as the degradation efficiency improved, the magnitude of the heat generation effect also improved. Hence, the spectral variations observed in our lab‐around‐microfiber sensor demonstrated the consistent nature of both the catalytic reaction process and the associated thermal effects.

Surface chemical and thermal events provide the key parameters of photoelectrocatalysts. This operando decoding provides reliable information for catalytic effect evaluation and catalytic mechanism exploration. It is crucial for understanding the photoelectrocatalysis mechanism and further improving its performance. The proposed fiber optic detection method requires minimal catalyst, rendering it suitable for real‐time and in situ monitoring of catalytic reactions with limited catalyst quantities. In the future, this approach can be integrated with existing catalytic monitoring techniques to address the limitations associated with bulky instrumentation and lack of real‐time in situ monitoring. Moreover, it holds potential as a portable tool for on‐site assessment of catalyst reaction parameters.

## 3 Conclusion

In summary, we have successfully demonstrated the feasibility of a lab‐around‐microfiber sensor for simulating photocatalytic reactions on the surface of optical fibers, as well as in situ detection of the concentration of the reactants and heat production on the catalyst surface. The sensor could distinguish the distinct excitation effects of irradiation and voltage on catalytic activity during photoelectrocatalysis; thus, the primary light‐induced excitation effect and the secondary voltage‐induced excitation effect were elucidated. It could also be used to evaluate the impacts of different light wavelengths on catalytic efficiency, thereby providing the correlation between the catalyst's internal structure and its catalytic performance. Moreover, our sensor enabled the elucidation of pollutant degradation and catalytic heat generation processes occurring at the microscale on the catalyst surface; thereby, the synchronized occurrence of pollutant degradation and catalytic thermal effects was determined. This approach provided a basic understanding of the catalytic process and mechanism by addressing an important deficiency in the current catalytic process and heat monitoring methods. It also provided a new method for the operando decoding of physical and chemical events at the submicron scale adjacent to the sensor.

## 4 Experimental Section

### Materials and Reagents

All chemicals were purchased without any further purification. A tetracycline solution was used as the pollutant model. It was diluted with water (100 mL) to prepare a 0.2 mg mL^−1^ solution.

### Synthesis of Bi_2_O_3_

The Bi_2_O_3_ nanospheres were synthesized following a prior study.^[^ ^49^ ^]^ In brief, Bi(NO_3_)_3_·5H_2_O (0.5 g) and glucose (1 mmol) were dissolved in 15 mL of ethylene glycol. The mixture was stirred and placed in a Teflon‐lined stainless autoclave and heated. Finally, the product was collected and washed.

### Synthesis of Bi_2_O_3_‐UCNPs

Bi(NO_3_)_3_·5H_2_O (0.5 g) and glucose (1 mmol) were dissolved in 15 mL of ethylene glycol. Subsequently, Yb(NO_3_)_3_·5H_2_O and Tm(NO_3_)_3_·5H_2_O were incorporated into the mixture, and the mixture was stirred at ambient temperature for a duration of 1 h, followed by ultrasonic treatment in a water bath for 20 min. The mixture was placed in a 50 mL Teflon‐lined stainless autoclave, and heated at 160 °C for 18 h. The product was cooled to ambient temperature through natural convection. Finally, the product was collected and washed.

The added addition amounts of Yb(NO_3_)_3_·5H_2_O and Tm(NO_3_)_3_·5H_2_O were as follows: Bi_2_O_3_‐ UCNP0.5: 0.05 g of Yb(NO_3_)_3_·5H_2_O and 0.0075 g of Tm(NO_3_)_3_·5H_2_O; Bi_2_O_3_‐UCNP1: 0.1 g of Yb(NO_3_)_3_·5H_2_O and 0.015 g of Tm(NO_3_)_3_·5H_2_O; and Bi_2_O_3_‐UCNP2: 0.2 g of Yb(NO_3_)_3_·5H_2_O and 0.03 g of Tm(NO_3_)_3_·5H_2_O.

### Characterization

The morphology of the catalysts was investigated using TEM (JEM‐2100F, JEOL) and SEM (ULTRA55, ZEISS). The XRD patterns were obtained using a PANalytical B.V. Empyrean diffractometer equipped with Cu Kα radiation. XPS was performed with a Thermo Fisher Scientific K‐AlPHA system, utilizing single Al k‐α radiation (1486.68 eV, 12 kV). The UV–vis–IR spectra were measured using a spectrophotometer (Shimadzu UV‐2550).

### Silica Optical Microfiber Fabrication

The fabrication of the silica optical microfibers was achieved through the flame‐heating stretching method.^[^ ^25^ ^]^ The double‐cladding single‐mode fiber (UV‐INT‐PREMIUM, 100536, CorActive High‐Tech Inc.) was gradually elongated and stretched to reduce its diameter as the hydrogen flame expanded to a width of 5 mm. Subsequently, a cone shape was formed to attain a microfiber with a diameter measuring 7.9 µm.

### Nanointerface Functionalization of Silica Microfibers

The silica microfiber was hydroxylated and aminated according to the previous work.^[^ ^40^ ^]^ The catalysts were amino‐modified in accordance with previous research.^[^ ^16^ ^]^ After, 1% glutaraldehyde was used to modify the microfiber surface. The microfiber was immersed in a catalyst dispersion (0.05 mmol mL^−1^) to form the lab‐around microfiber sensor. Finally, the lab‐aroundmicrofiber sensor was washed with deionized water and dried.

### Experimental Setup

The optical signal of the optical fiber was simulated by using a broadband light source (BBS), and subsequently recorded with a spectrum analysis (OSA). A custom light‐emitting diode (LED) with a wavelength range of 275–420 nm and a power of 20 W was used as the visible light source for photocatalysis. A pump laser with a wavelength of 980 nm and a power of 150 mW was coupled into the fiber as the near‐infrared light source for photocatalysis. A cyclic scanning voltage ranging from 0.5 to 2 V was used as the electric excitation source for the photoelectrocatalysis.

### Statistical Analysis

The data were presented as the mean ± standard deviation (SD) and analyzed by the GraphPad Prism 7. All experiments were conducted at least three times to ensure reproducibility.

## Conflict of Interest

The authors declare no conflict of interest.

## Author Contributions

Y.H., C.M., and J.L. contributed equally to this work. Y. H. designed experiments, performed data interpretation, and wrote the manuscript. B.O.G. conceived the study and supervised the experiment. C. M. and J. L. carried out the experiments and analyzed the data, with assistance from J.W. and P.C.

## Supporting information

Supporting Information

## Data Availability

The data that support the findings of this study are available from the corresponding author upon reasonable request.
