# Supplementary material for: Operando Decoding of Surface Chemical and Thermal Events in Photoelectrocatalysis via a Lab‐Around‐Microfiber Sensor
Source: Adv Sci (Weinh). 2024 Apr 30;11(26):2310264. doi: 10.1002/advs.202310264 (PMC11234440; doi:10.1002/advs.202310264)
Supplement: Supplementary file 1 — Supporting Information [file ADVS-11-2310264-s001.pdf]

ADVANCED SCIENCE
Open Access

Supporting Information

for *Adv. Sci.*, DOI 10.1002/advs.202310264

Operando Decoding of Surface Chemical and Thermal Events in Photoelectrocatalysis via a Lab-Around-Microfiber Sensor

Yunyun Huang, Caini Mou, Jiaxuan Liang, Jiaxin Wan, Pengwei Chen and Bai-Ou Guan**

Operando Decoding of Surface Chemical and Thermal Events in Photoelectrocatalysts via a Lab-Around-Microfiber Sensor

Yunyun Huang, †* *Caini Mou,*† *Jiaxuan Liang,*† *Jiaxin Wan, Pengwei Chen, and Bai-Ou Guan**

Guangdong Provincial Key Laboratory of Optical Fiber Sensing and Communications, Institute of Photonics Technology, Jinan University, Guangzhou 511143, China

E-mail: Yunyun Huang, email: thuangyy6@jnu.edu.cn

Bai-Ou Guan, email: tguanbo@jnu.edu.cn

Content

Supplementary figures

Figure S1. Tapering process of the optical microfiber.

Figure S2. Optical microscopy photographs of the uniform region and transition region of the microfiber.

Figure S3. TEM images.

Figure S4. High-resolution XPS.

Figure S5. Energy-dispersive spectroscopy (EDS) result of Bi_2O_3.

Figure S6. EDS result of Bi_2O_3-UCNP0.5..

Figure S7. EDS result of Bi_2O_3-UCNP1.

Figure S8. EDS result of Bi_2O_3-UCNP2.

Figure S9. Band gap energy of Bi_2O_3.

Figure S10. Upconversion PL spectra of the catalysts under 980 nm excitation.

Figure S11. Transmission spectra of the lab around fiber fabrications.

Figure S12. Distribution statistics of Bi_2O_3 on microfiber surface.

Figure S13. Distribution statistics of Bi_2O_3-UCNPs0.5 on microfiber surface.

Figure S14. Distribution statistics of Bi_2O_3-UCNPs1 on microfiber surface.

Figure S15. Distribution statistics of Bi_2O_3-UCNPs2 on microfiber surface.

Figure S16. Distribution statistics of Bi_2O_3 on FBG surface.

Figure S17. Distribution statistics of Bi_2O_3-UCNPs0.5 on FBG surface.

Figure S18. Distribution statistics of Bi_2O_3-UCNPs1 on FBG surface.

Figure S19. Distribution statistics of Bi_2O_3-UCNPs2 on FBG surface.

Figure S20. Optical stability of the lab around microfiber in aqueous solution.

Figure S21. Measured transmission spectra when tetracycline molecules were adsorbed onto the catalyst surfaces.

Figure S22. Measured transmission spectra when catalysts around fibers were under visible light and voltage.

Figure S23. Wavelength shifts in the transmission fringe when catalysts around fibers were

under visible light and voltage.

Figure S24. UV-Visible absorption spectra of tetracycline during the catalytic process and corresponding degradation efficiency.

Figure S25. Wavelength shifts in the transmission fringe when catalysts around fibers were under NIR light and voltage.

Figure S26. Wavelength shifts in the transmission fringe when catalysts around microFBGs.

Figure S27. Wavelength shifts of the microFBG versus time (subtraction results) and the corresponding temperature rises in the photoelectrocatalysis process..

Figure S28. Wavelength shifts of the microFBG versus time in five degradation cycles of tetracycline over Bi_2O_3.

Figure S29. Mott-Schottky plots.

Figure S30. Relationship between spectral derivatives of the pollutant degradation process (($|\lambda_0|-|\triangle\lambda|)/|\lambda_0|$ over time) and derivatives of the temperature changes. Catalyst: Bi_2O_3-UCNP0.5..

Supplementary tables

Table S1. Element content of Bi_2O_3 obtained from the EDS result.

Table S2. Element content of Bi_2O_3-UCNP0.5 obtained from the EDS result.

Table S3. Element content of Bi_2O_3-UCNP1 obtained from the EDS result.

Table S4. Element content of Bi_2O_3-UCNP2 obtained from the EDS result.

Table S5. Distribution statistics of Bi_2O_3 calculated from Figure S6.

Table S6. Distribution statistics of Bi_2O_3-UCNPs0.5 calculated from Figure S7.

Table S7. Distribution statistics of Bi_2O_3-UCNPs1 calculated from Figure S8.

Table S8. Distribution statistics of Bi_2O_3-UCNPs2 calculated from Figure S9.

Table S9. Distribution statistics of Bi_2O_3 calculated from Figure S10.

Table S10. Distribution statistics of Bi_2O_3-UCNPs0.5 calculated from Figure S11.

Table S11. Distribution statistics of Bi_2O_3-UCNPs1 calculated from Figure S12.

Table S12. Distribution statistics of Bi_2O_3-UCNPs2 calculated from Figure S13.

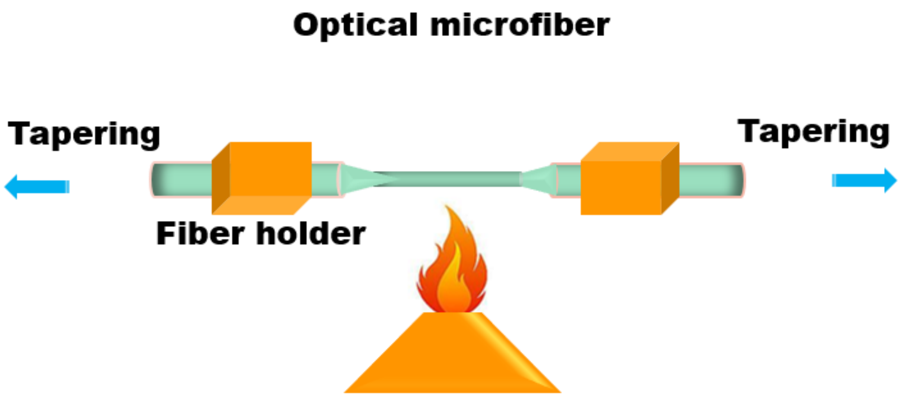

Figure S1. Tapering process of the optical microfiber.

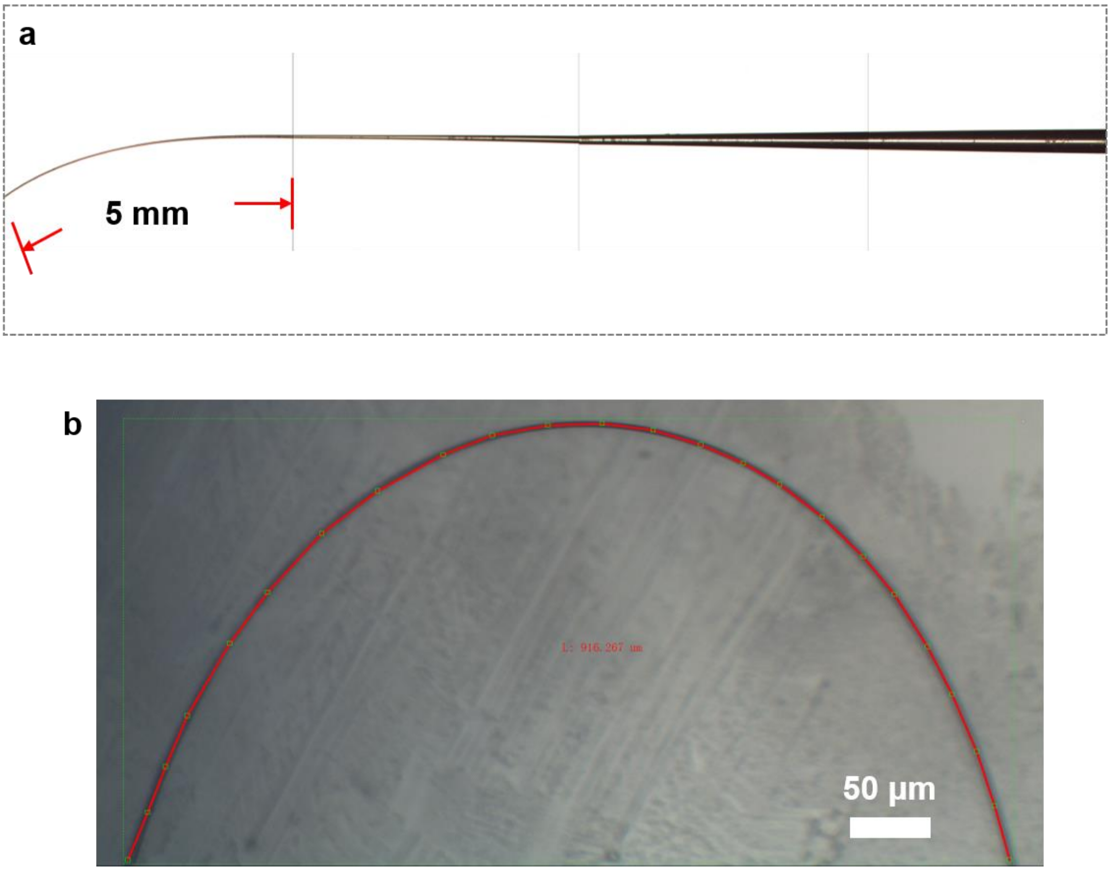

Figure S2. Optical microscopy photographs of the uniform region and transition region of the microfiber.

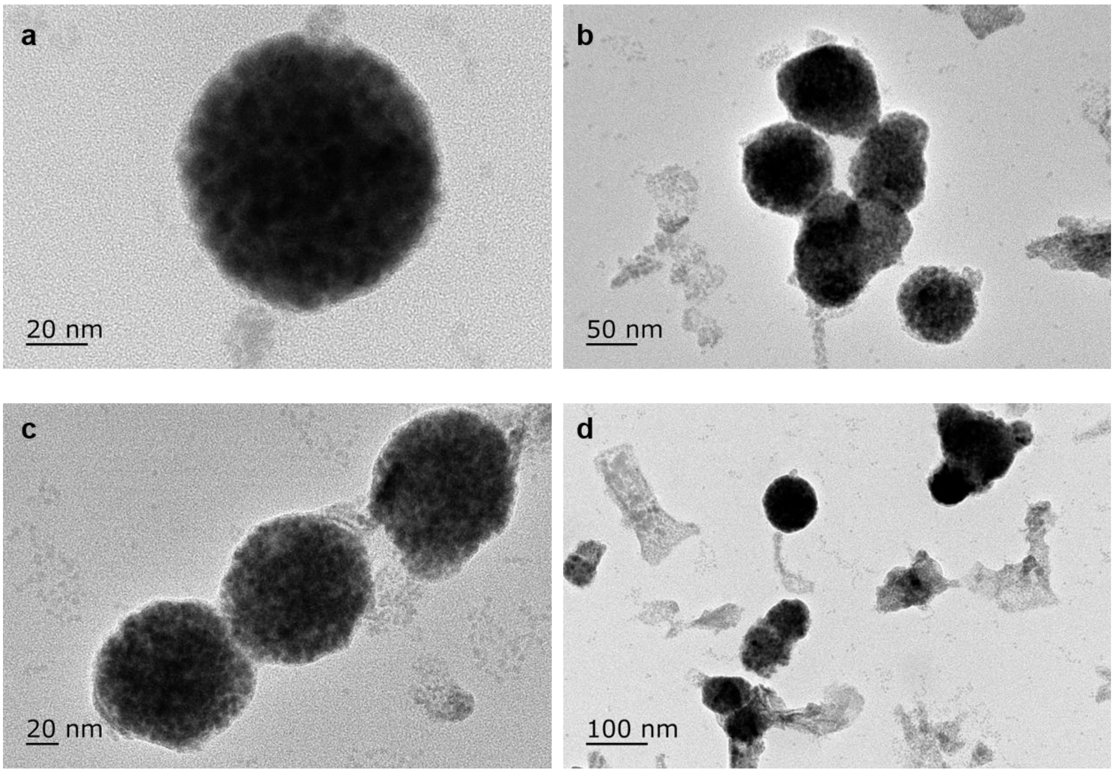

Figure S3. Transmission electron microscopy (TEM) images of (a) Bi_2O_3, (b) Bi_2O_3-UCNP0.5, (c) Bi_2O_3-UCNP-1, and (d) Bi_2O_3-UCNP-2.

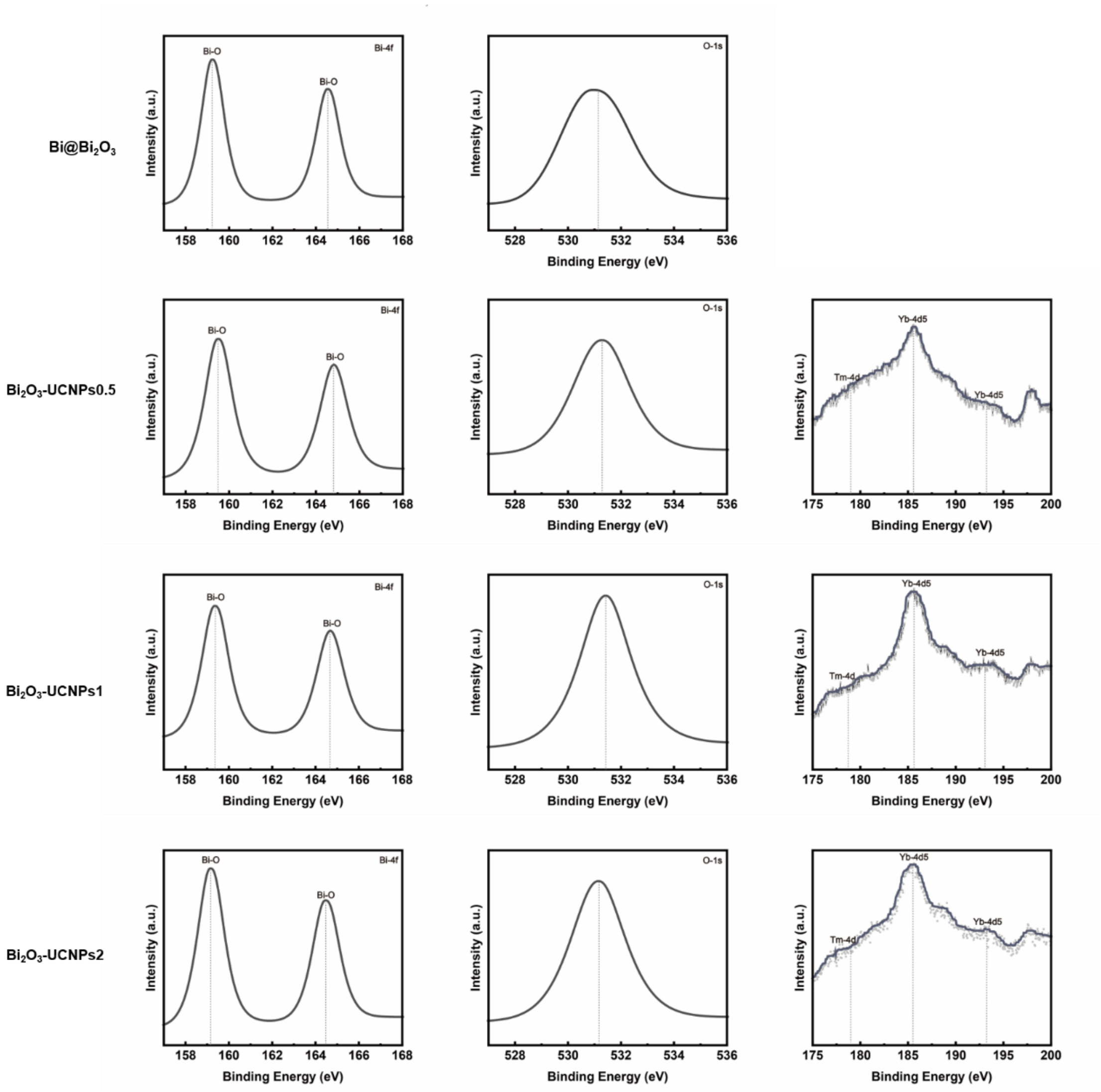

Figure S4. High-resolution X-ray photoelectron spectra (XPS) of Bi 4f, O 1s and Yb4d5 in Bi_2O_3 and Bi_2O_3-UCNPs.

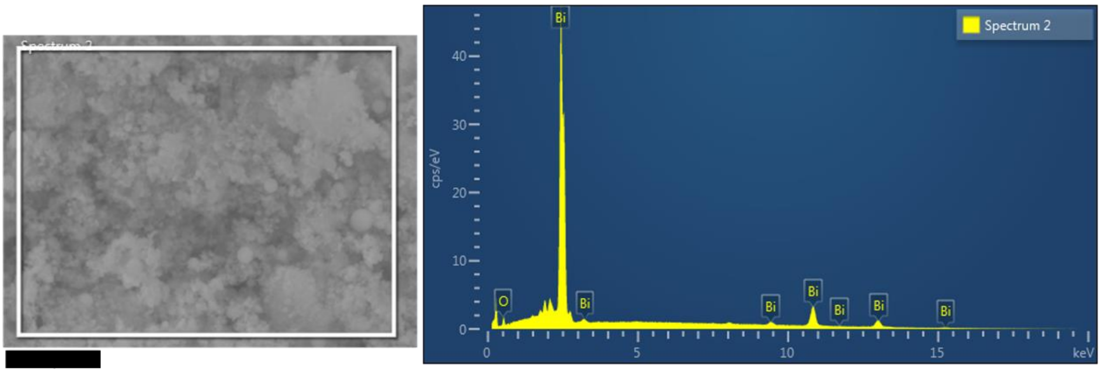

Figure S5. Energy-dispersive spectroscopy (EDS) result of Bi_2O_3.

Table S1. Element content of Bi_2O_3 obtained from the EDS result.

**Element**	**Wt (%)**
O	4.06
Bi	95.94

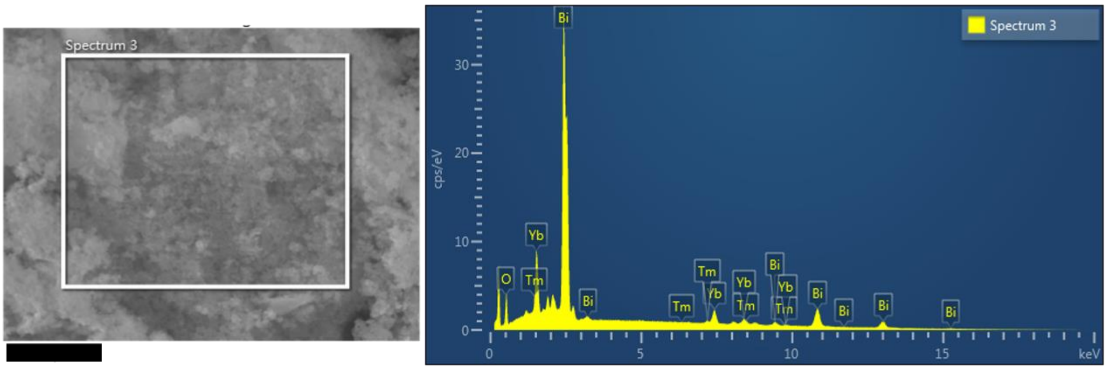

Figure S6. EDS result of Bi_2O_3-UCNP0.5.

Table S2. Element content of Bi_2O_3-UCNP0.5 obtained from the EDS result.

**Element**	**Wt (%)**
O	9.37
Bi	75.57
Yb	12.68
Tm	2.38

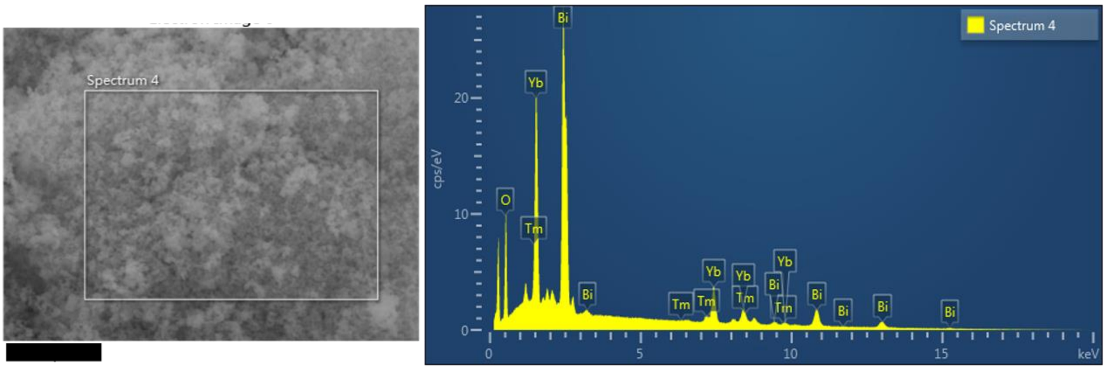

Figure S7. EDS result of Bi_2O_3-UCNP1.

Table S3. Element content of Bi_2O_3-UCNP1 obtained from the EDS result.

Element	Wt (%)
O	18.27
Bi	54.80
Yb	22.93
Tm	4.00

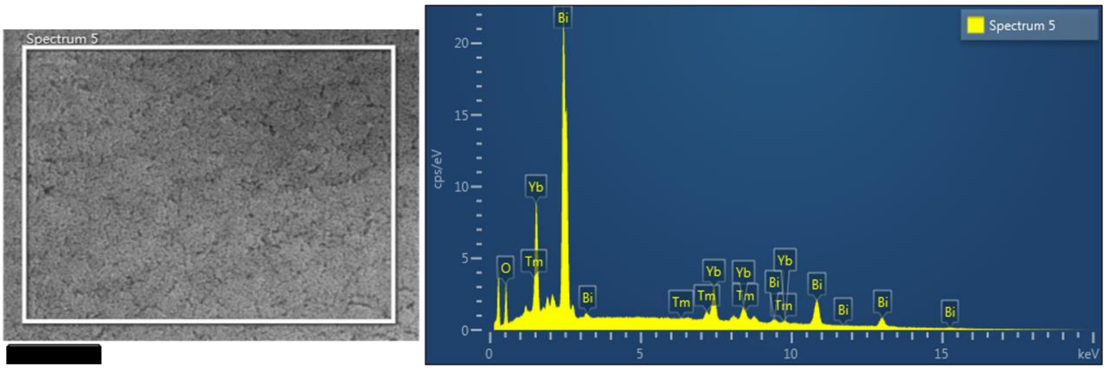

Figure S8. EDS result of Bi_2O_3-UCNP2.

Table S4. Element content of Bi_2O_3-UCNP2 obtained from the EDS result.

Element	Wt (%)
O	8.96
Bi	53.51

Yb	30.17
Tm	7.36

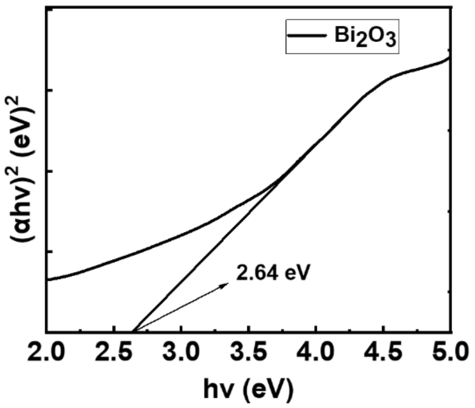

Figure S9. Band gap energy of Bi_2O_3.

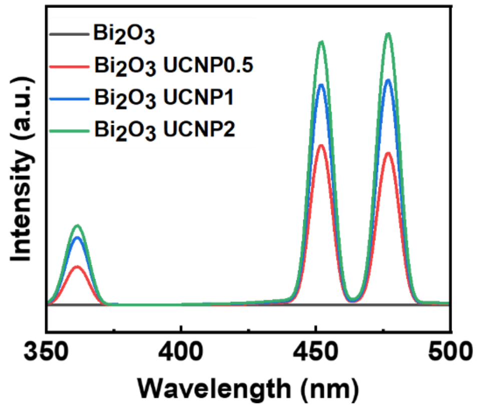

Figure S10. Upconversion PL spectra of the catalysts under 980 nm excitation. (The instrument's limitations restrict the measurement of wavelengths to a minimum of 350 nm.)

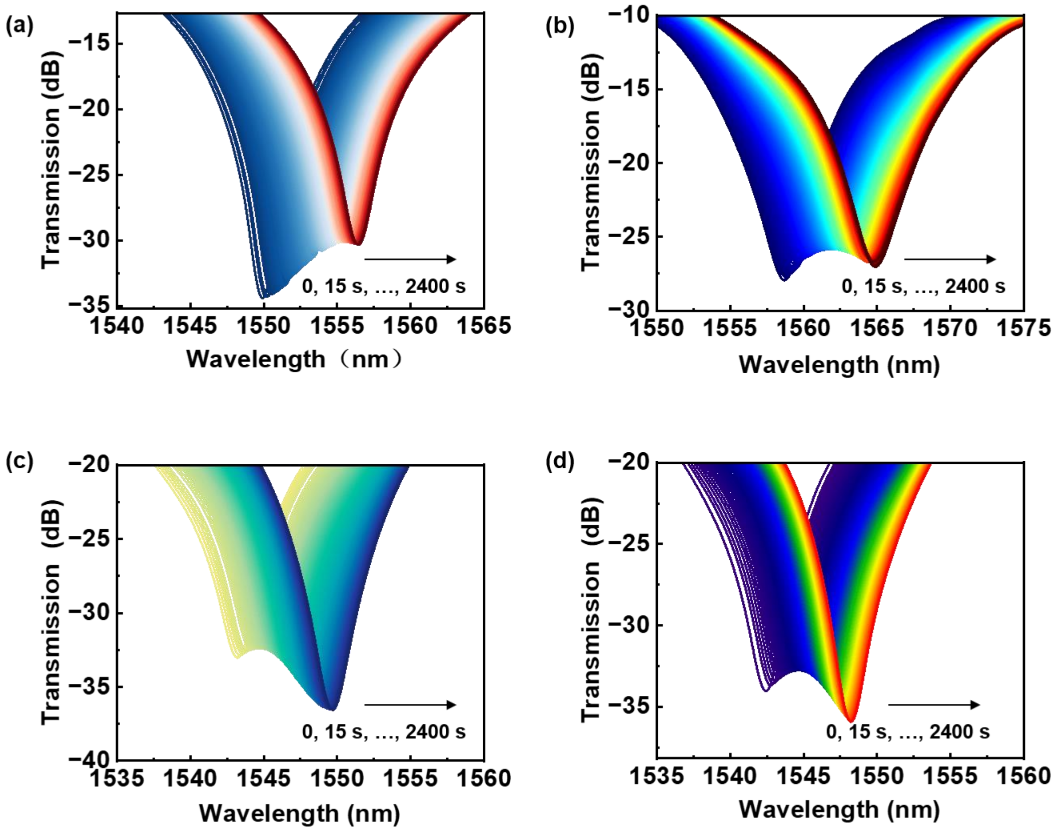

Figure S11. Transmission spectra of the lab around fiber fabrication of (a) Bi_2O_3, (b) Bi_2O_3-UCNPs0.5, (c) Bi_2O_3-UCNPs1, and (d) Bi_2O_3-UCNPs2.

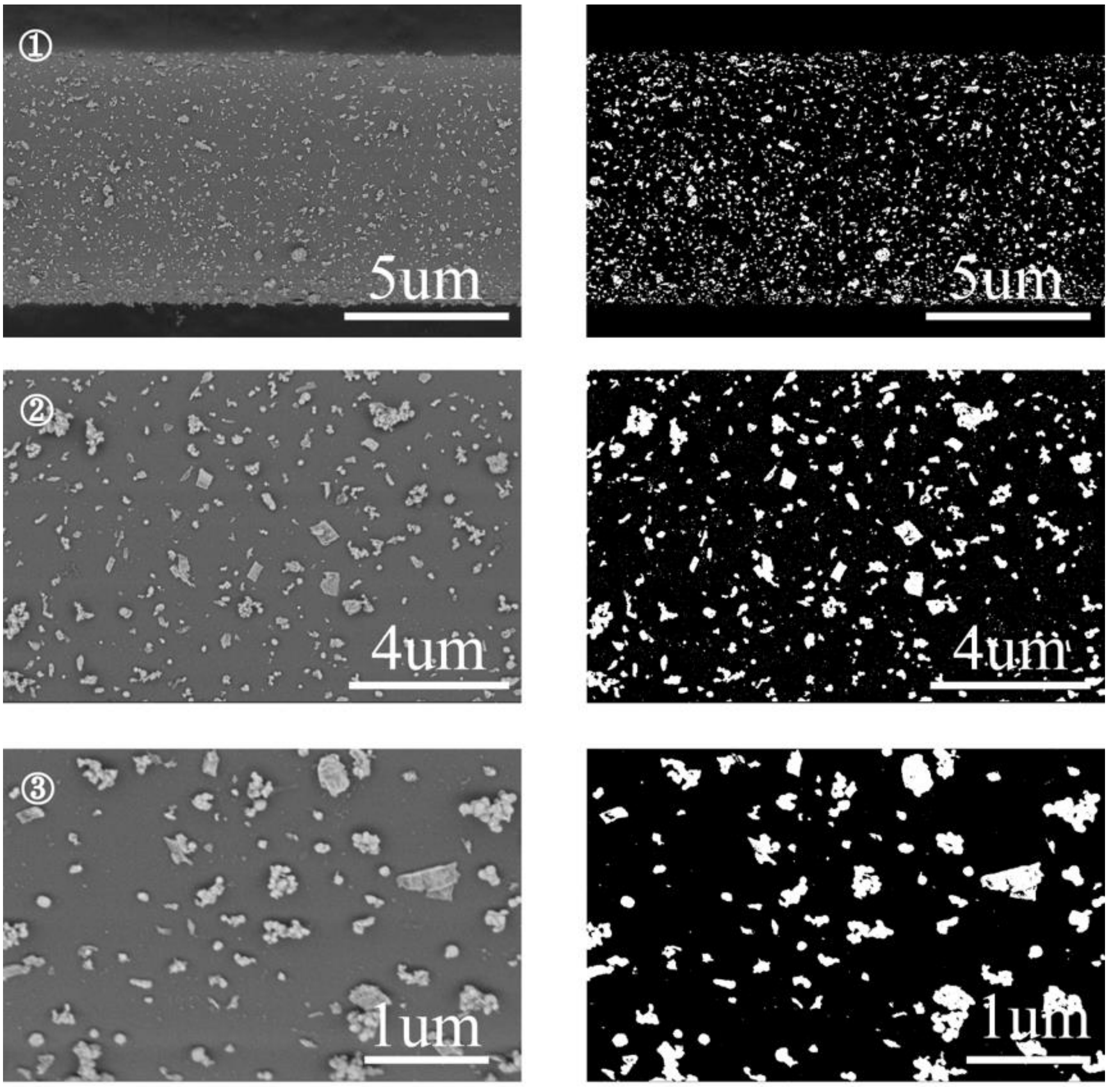

Figure S12. Distribution statistics of Bi_2O_3 on microfiber surface.

Table S5. Distribution statistics of $Bi@Bi_2O_3$ calculated from Figure S6 (mean ± SD, n = 3).

**Number**	**Bi_2O_3 (%)**
①	**11.42**
②	**11.06**
③	**11.9**
**Average**	**11.19±0.34**

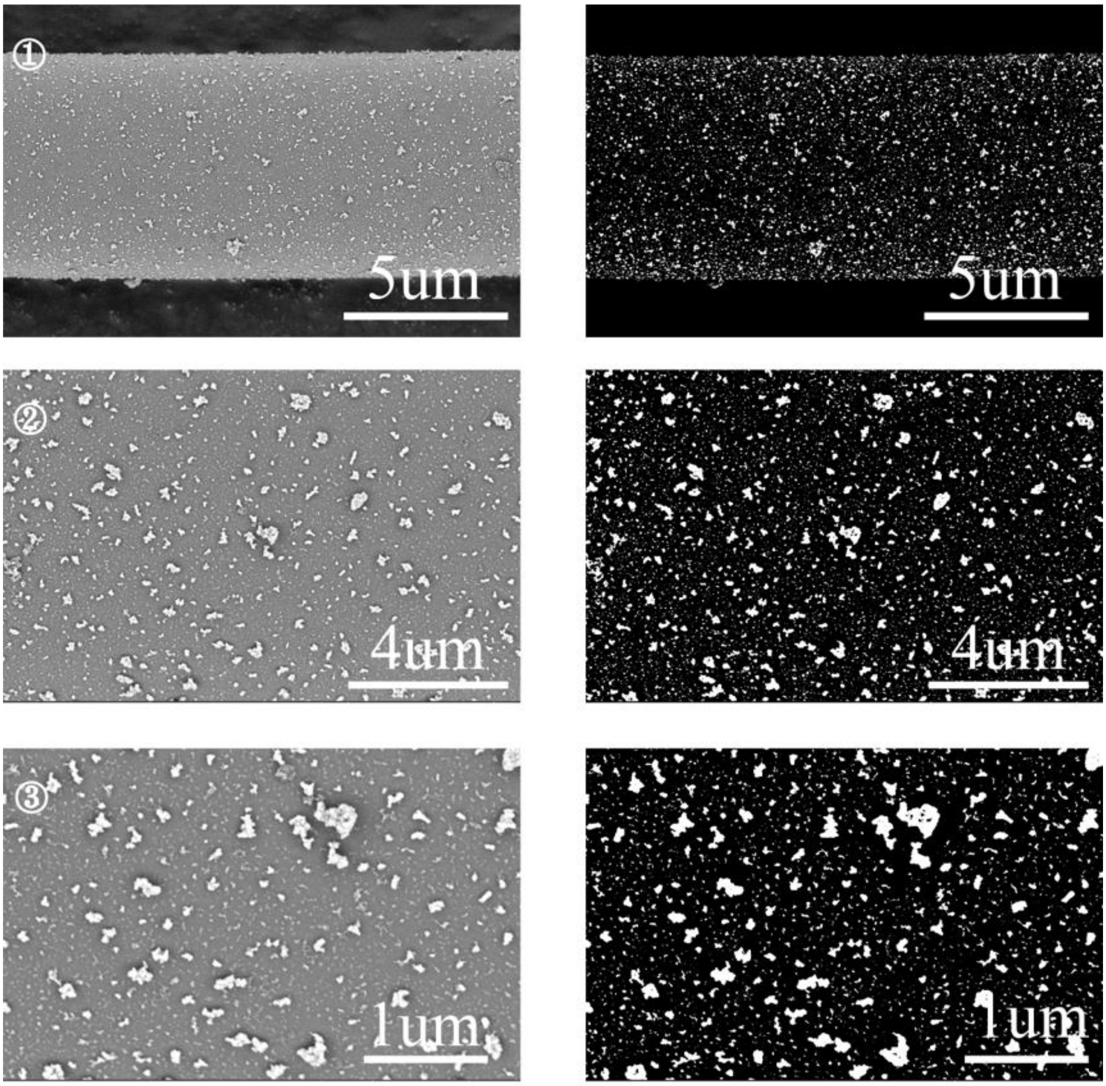

Figure S13. Distribution statistics of Bi_2O_3-UCNPs0.5 on microfiber surface.

Table S6. Distribution statistics of Bi_2O_3-UCNPs0.5 calculated from Figure S7 (mean ± SD, n = 3).

**Number**	**Bi_2O_3 UCNPs0.5 (%)**
①	**10.33**
②	**10.06**
③	**11.38**
**Average**	**10.59±0.57**

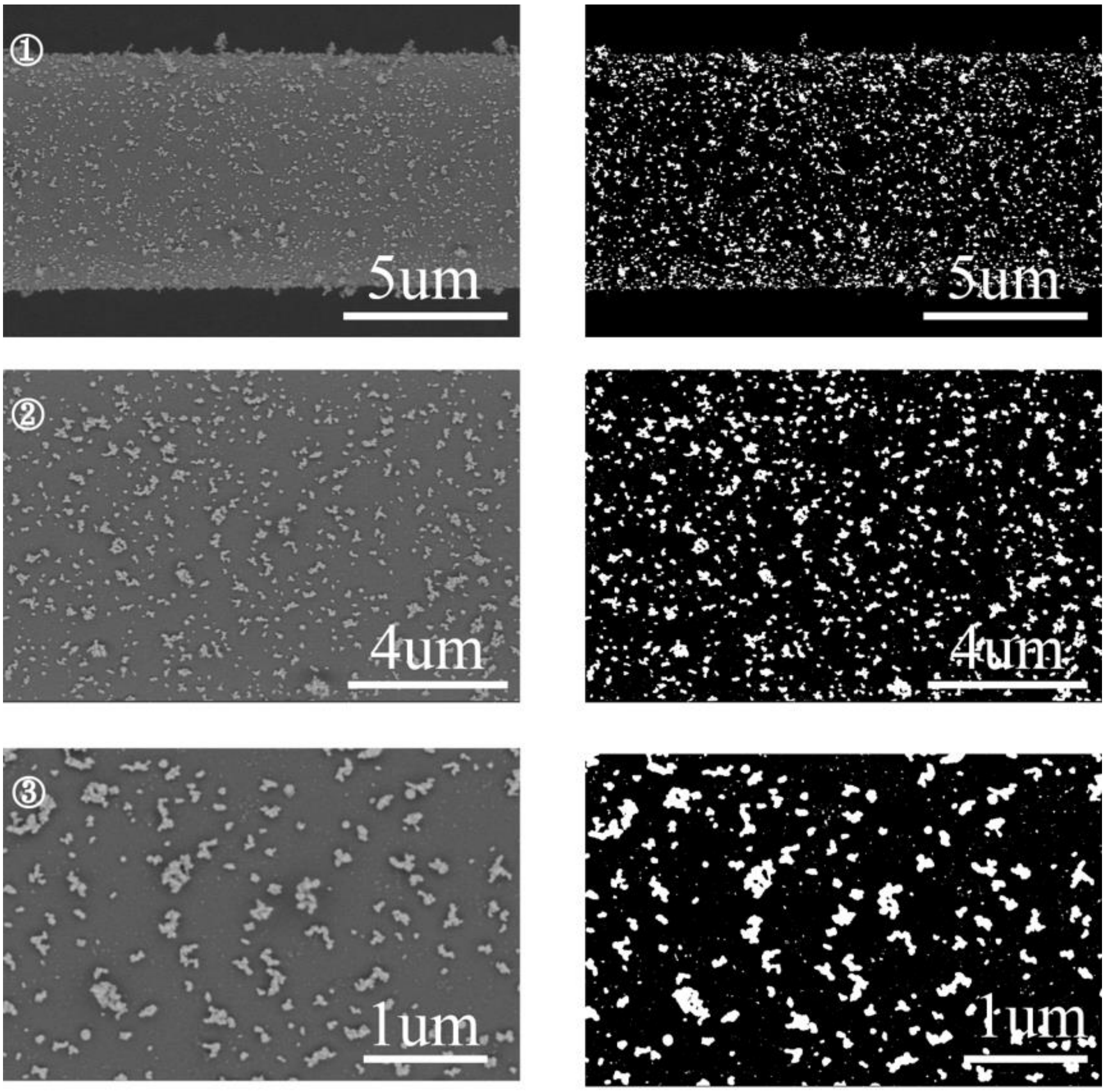

Figure S14. Distribution statistics of Bi_2O_3-UCNPs1 on microfiber surface.

Table S7. Distribution statistics of Bi_2O_3-UCNPs1 calculated from Figure S8 (mean ± SD, n = 3).

**Number**	**Bi_2O_3 UCNPs 1 (%)**
①	**12.11**
②	**12.71**
③	**12.98**
**Average**	**12.6±0.36**

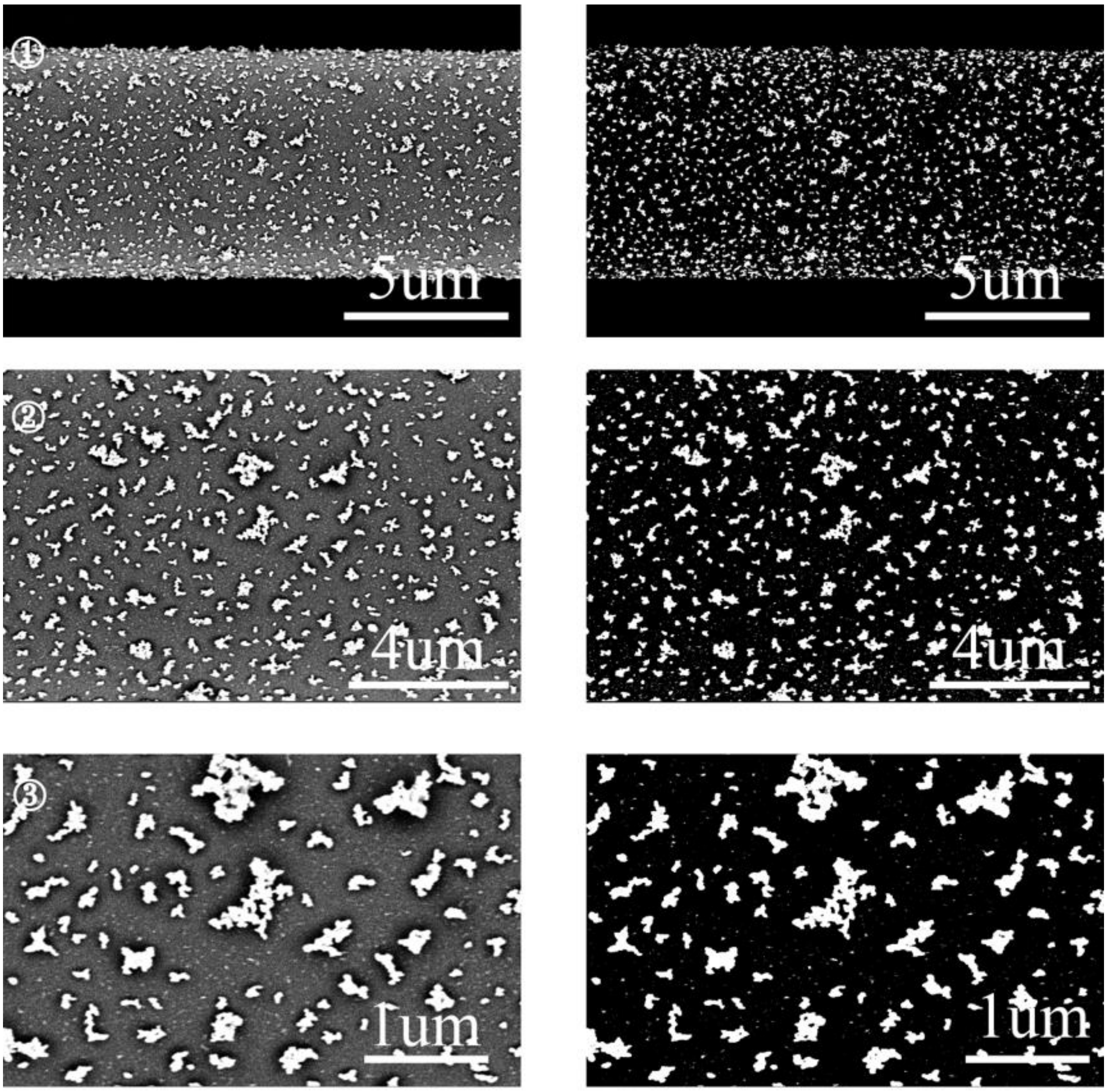

Figure S15. Distribution statistics of Bi_2O_3-UCNPs2 on microfiber surface.

Table S8. Distribution statistics of Bi_2O_3-UCNPs2 calculated from Figure S9 (mean ± SD, n = 3).

**Number**	**Bi_2O_3 UCNPs 2 (%)**
①	**12.8**
②	**12.71**
③	**12.98**
**Average**	**12.83±0.013**

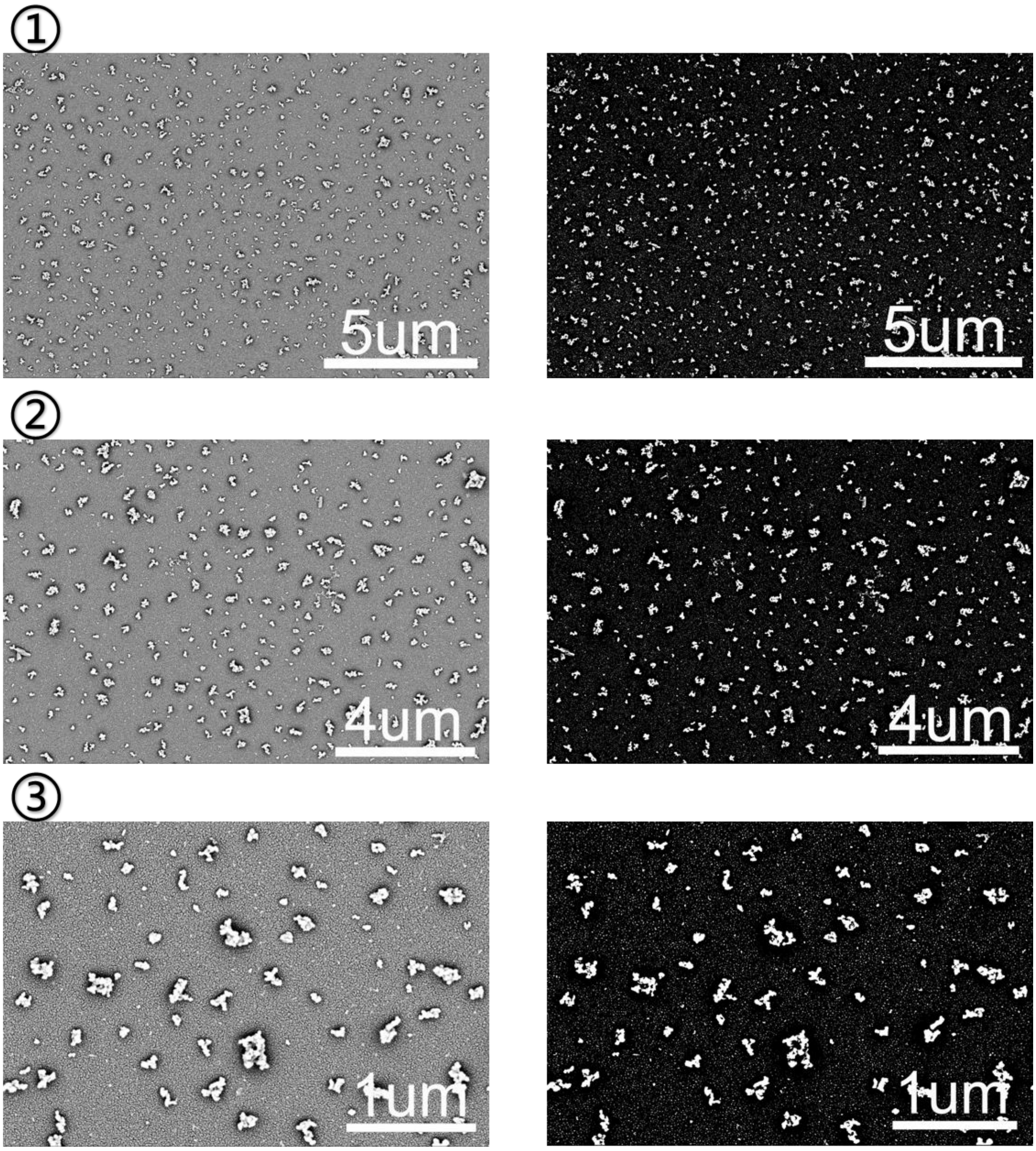

Figure S16. Distribution statistics of Bi_2O_3 on fiber Bragg grating (FBG) surface.

Table S9. Distribution statistics of Bi_2O_3 calculated from Figure S10 (mean ± SD, n = 3).

**Number**	**Bi_2O_3 (%)**
①	10.38
②	9.4
③	10.97
**Average**	10.25**±0.65**

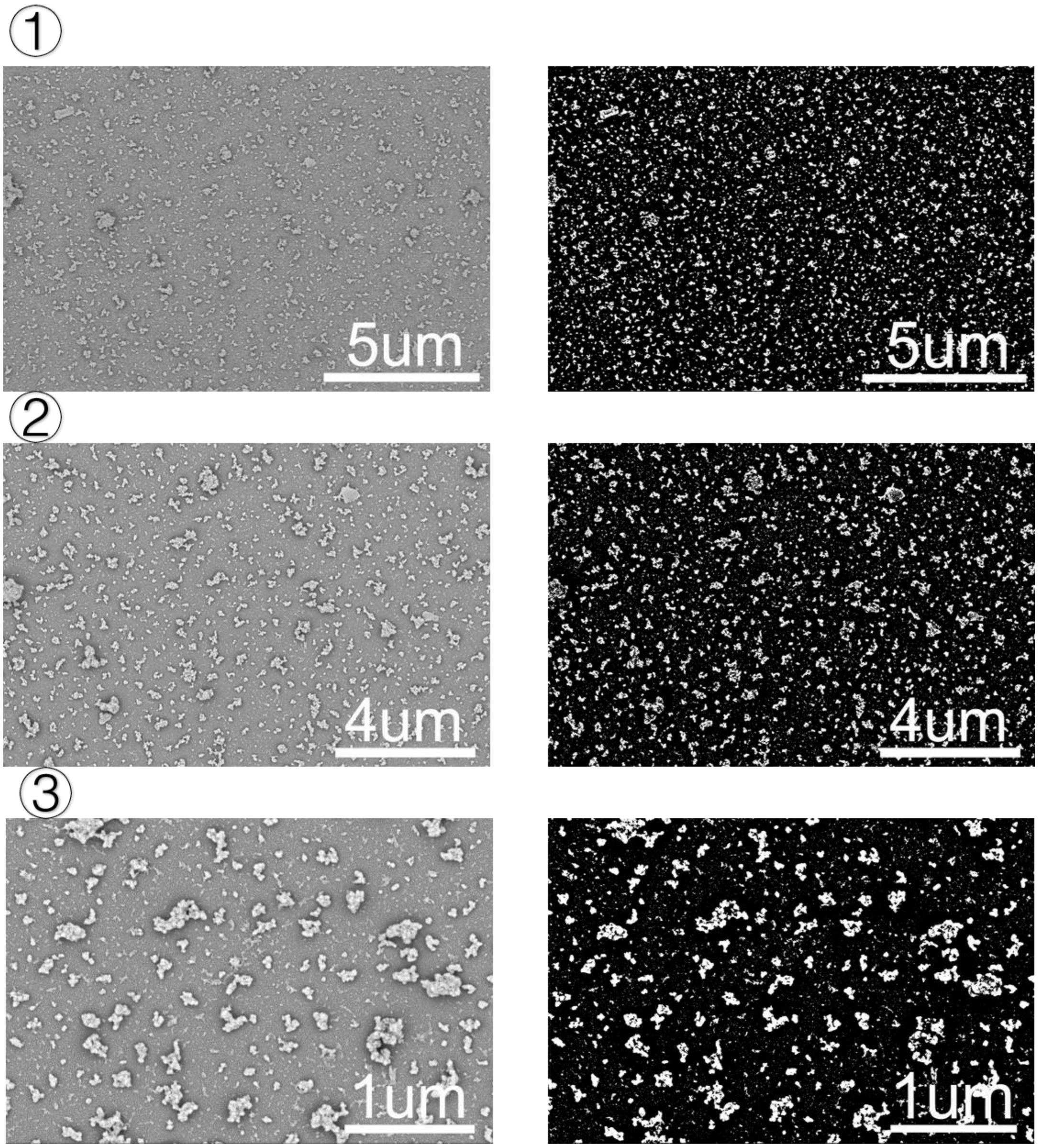

Figure S17. Distribution statistics of Bi_2O_3-UCNPs0.5 on FBG surface.

Table S10. Distribution statistics of Bi_2O_3-UCNPs0.5 calculated from Figure S11 (mean ± SD, n = 3).

**Number**	**Bi_2O_3 UCNPs0.5 (%)**
①	11.28
②	11.47
③	11.91

**Average**	11.55**±0.070**

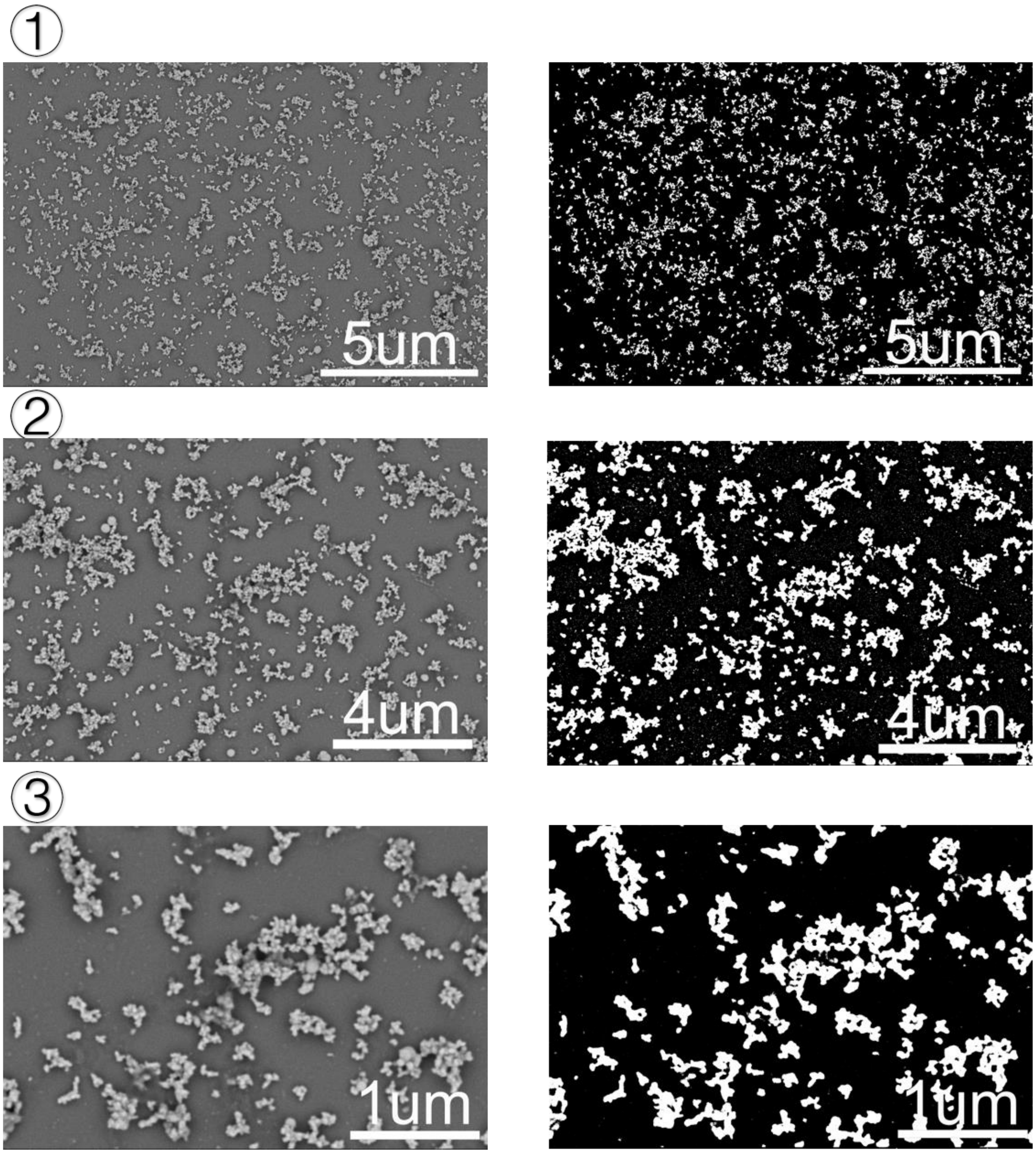

Figure S18. Distribution statistics of Bi_2O_3-UCNPs1 on FBG surface.

Table S11. Distribution statistics of Bi_2O_3-UCNPs1 calculated from Figure S12 (mean ± SD, n = 3).

**Number**	**Bi_2O_3 UCNPs1 (%)**
①	12.55
②	12.69

③	11.9
**Average**	12.38**±0.12**

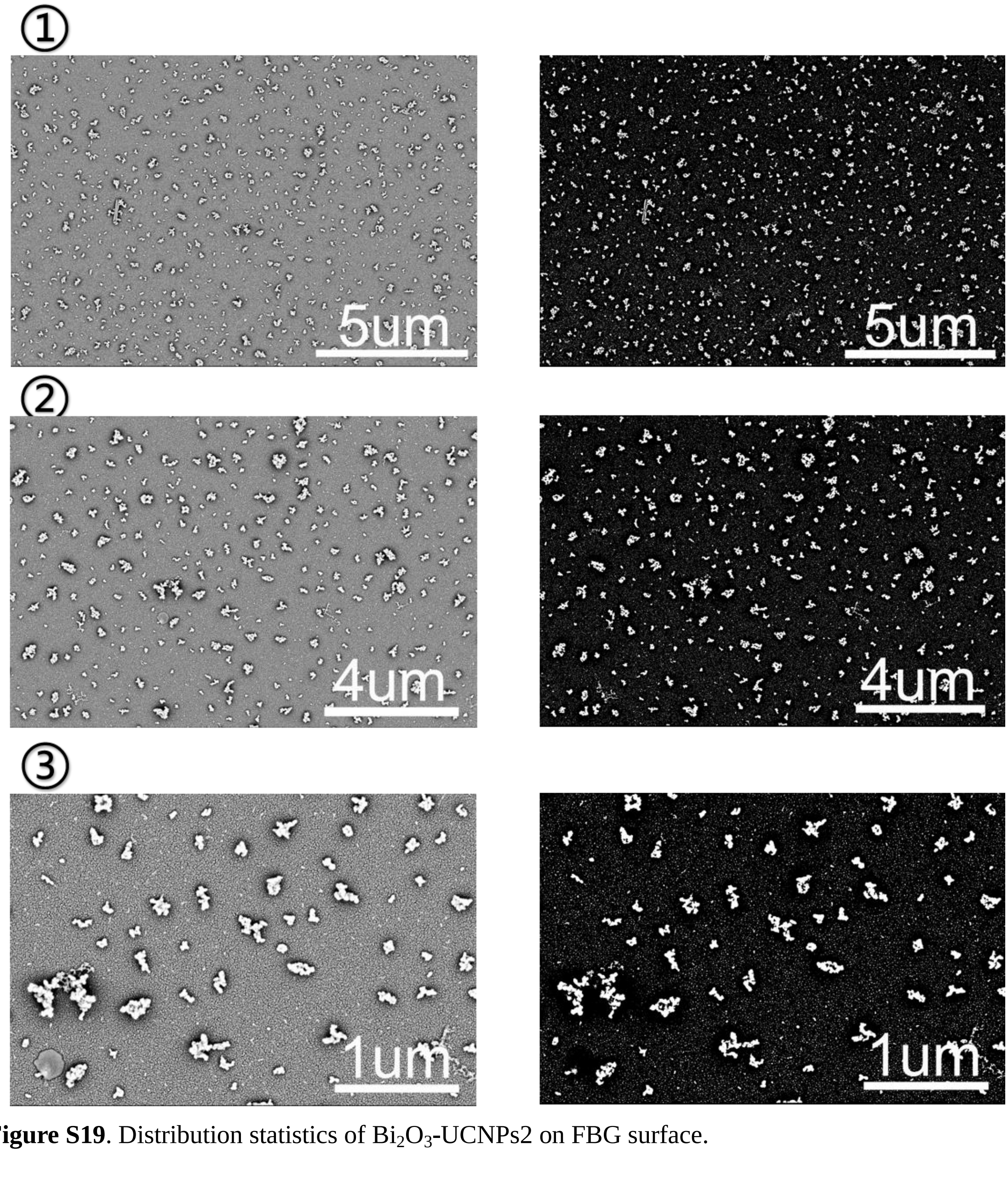

Figure S19. Distribution statistics of Bi_2O_3-UCNPs2 on FBG surface.

Table S12. Distribution statistics of Bi_2O_3-UCNPs2 calculated from Figure S13 (mean ± SD, n = 3).

**Number**	**Bi_2O_3 UCNPs2 (%)**
①	11.08

②	10.78
③	11.52
**Average**	11.13**±0.33**

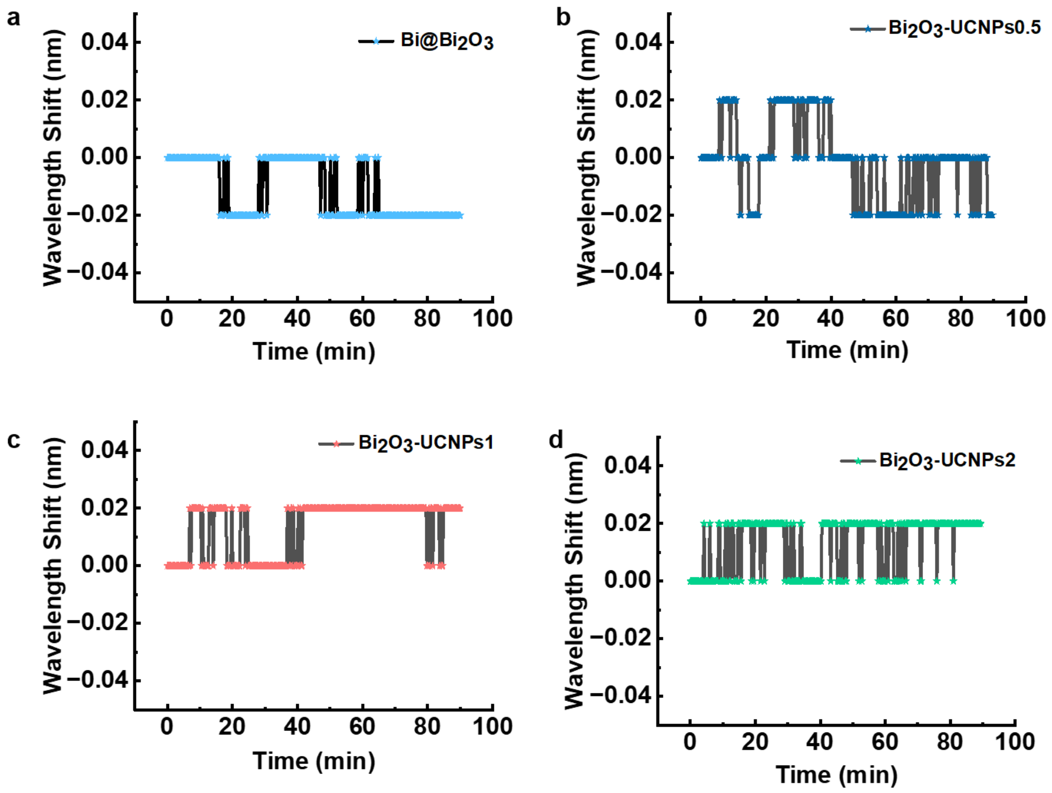

Figure S20. Optical stability of the lab around microfiber in aqueous solution. (a) Bi_2O_3, (b) Bi_2O_3-UCNP0.5, (c) Bi_2O_3-UCNP1, and (d) Bi_2O_3-UCNP2.

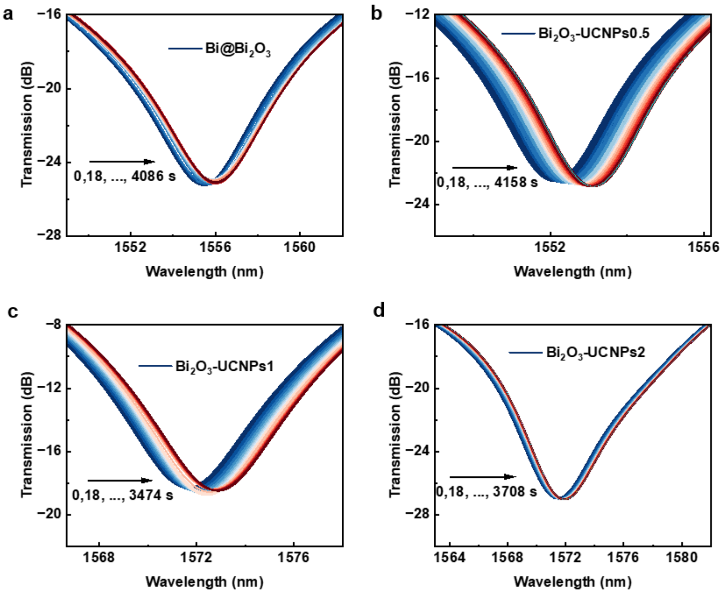

Figure S21. Measured transmission spectra when tetracycline molecules were adsorbed onto the catalyst surfaces. (a) Bi_2O_3-fiber, (b) Bi_2O_3-UCNP0.5-fiber, (c) Bi_2O_3-UCNP1-fiber, and (d) Bi_2O_3-UCNP2-fiber.

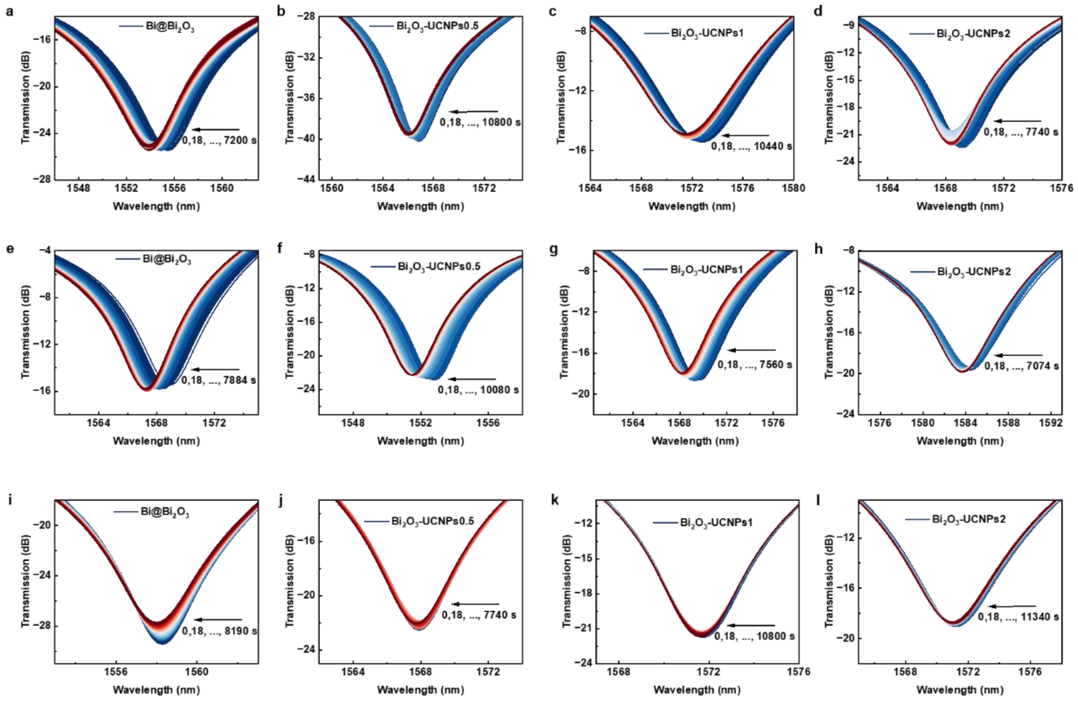

Figure S22. Measured transmission spectra when catalysts around fibers were under (a-d) visible light + voltage, (e-h) visible light, and (i-l) voltage. (**a, e, i** Bi_2O_3, **b, f, j** Bi_2O_3-UCNP0.5, **c, g, k** Bi_2O_3-UCNP1, **d, h, l** Bi_2O_3-UCNP2.)

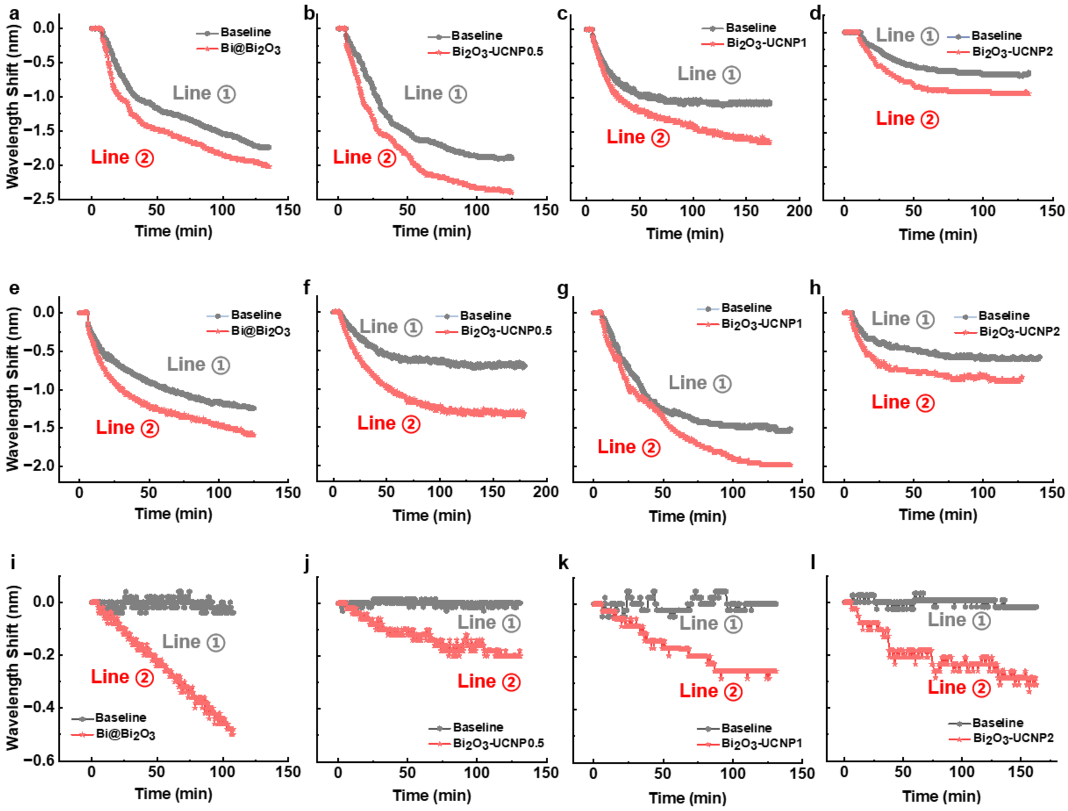

Figure S23. Wavelength shifts in the transmission fringe when catalysts around fibers were under (a-d) visible light + voltage, (e-h) visible light, and (i-l) voltage (line ②) and the corresponding baselines (line ①). (**a, e, i** Bi_2O_3, **b, f, j** Bi_2O_3-UCNP0.5, **c, g, k** Bi_2O_3-UCNP1, **d, h, l** Bi_2O_3-UCNP2; Line ① was recorded when the lab around fiber was under the simulation without pollutant.)

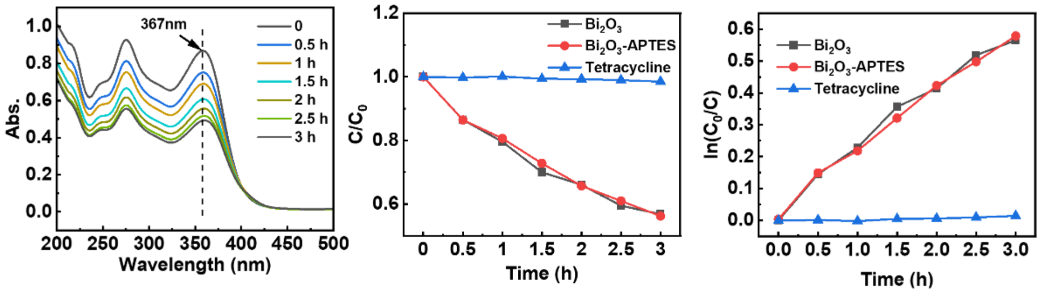

Figure S24. UV-Visible absorption spectra of tetracycline during the catalytic process and corresponding degradation efficiency (catalyst: APTES-treated and untreated Bi_3O_2).

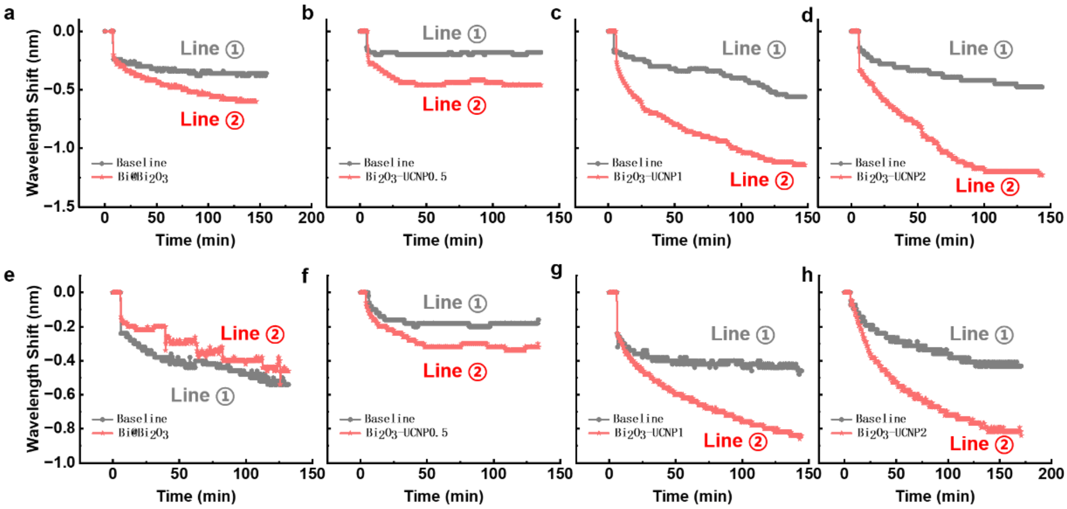

Figure S25. Wavelength shifts in the transmission fringe when catalysts around fibers were under (a-d) NIR light + voltage, (e-h) NIR light (line ②) and the corresponding baselines (line ①). (**a, e** Bi_2O_3, **b, f** Bi_2O_3-UCNP-0.5, **c, g** Bi_2O_3-UCNP-1, **d, h** Bi_2O_3-UCNP-2; Line ① was recorded when the lab around fiber was under the simulation without pollutant.)

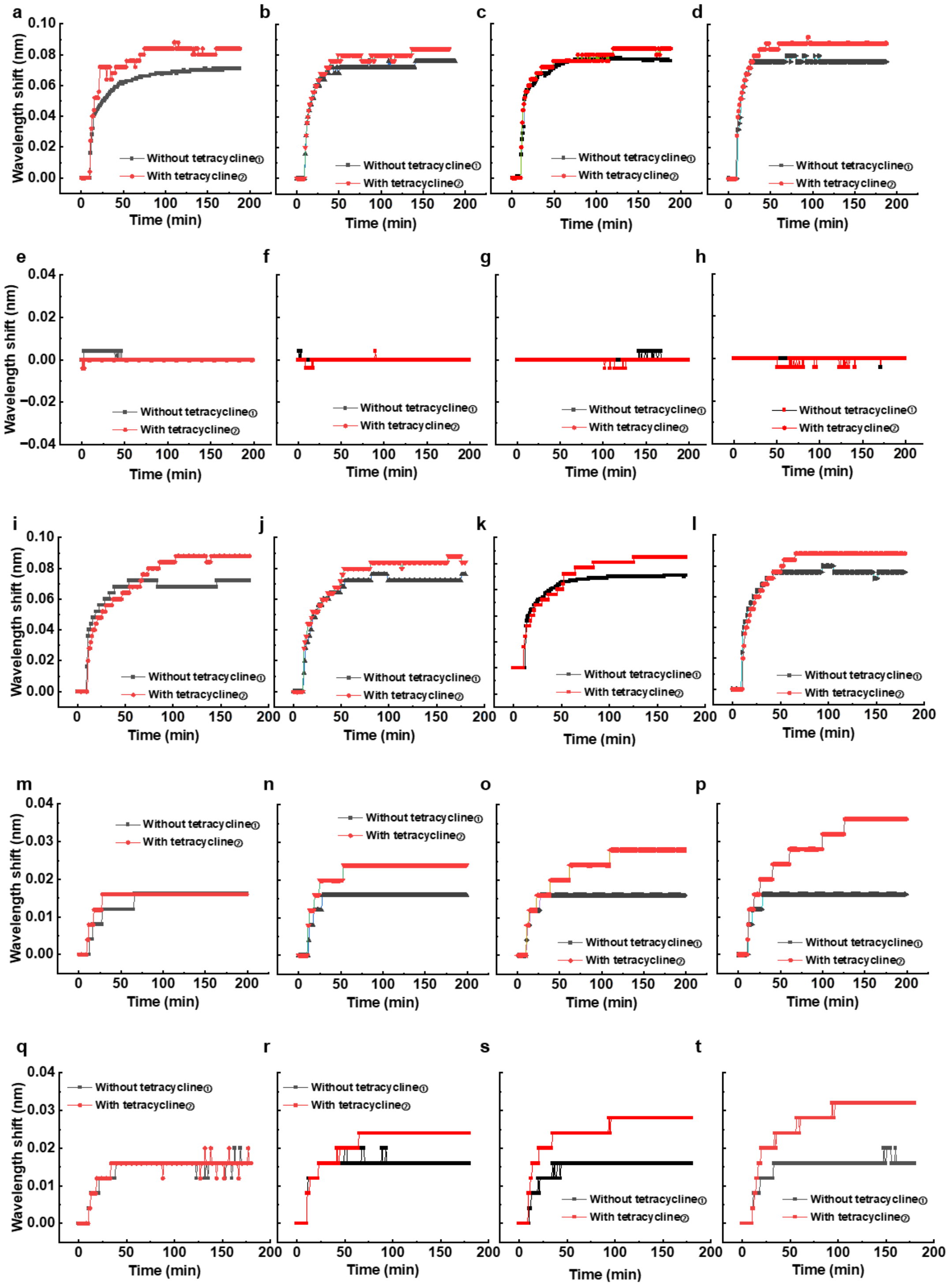

Figure S26. Wavelength shifts in the transmission fringe when catalysts around microFBGs were under **a-d** visible light, **e-h** voltage, **i-l** visible light + voltage, **m-p** NIR light, and **q-t** NIR light + voltage (line ②) and the corresponding baselines (line ①). (**a, e, i, m, q** Bi_2O_3, **b, f, j, n, r** Bi_2O_3-UCNP-0.5, **c, g, k, o, s** Bi_2O_3-UCNP-1, **d, h, l, p, t** Bi_2O_3-UCNP-2; Line ① was recorded when the lab around fiber was under the simulation without pollutant.)

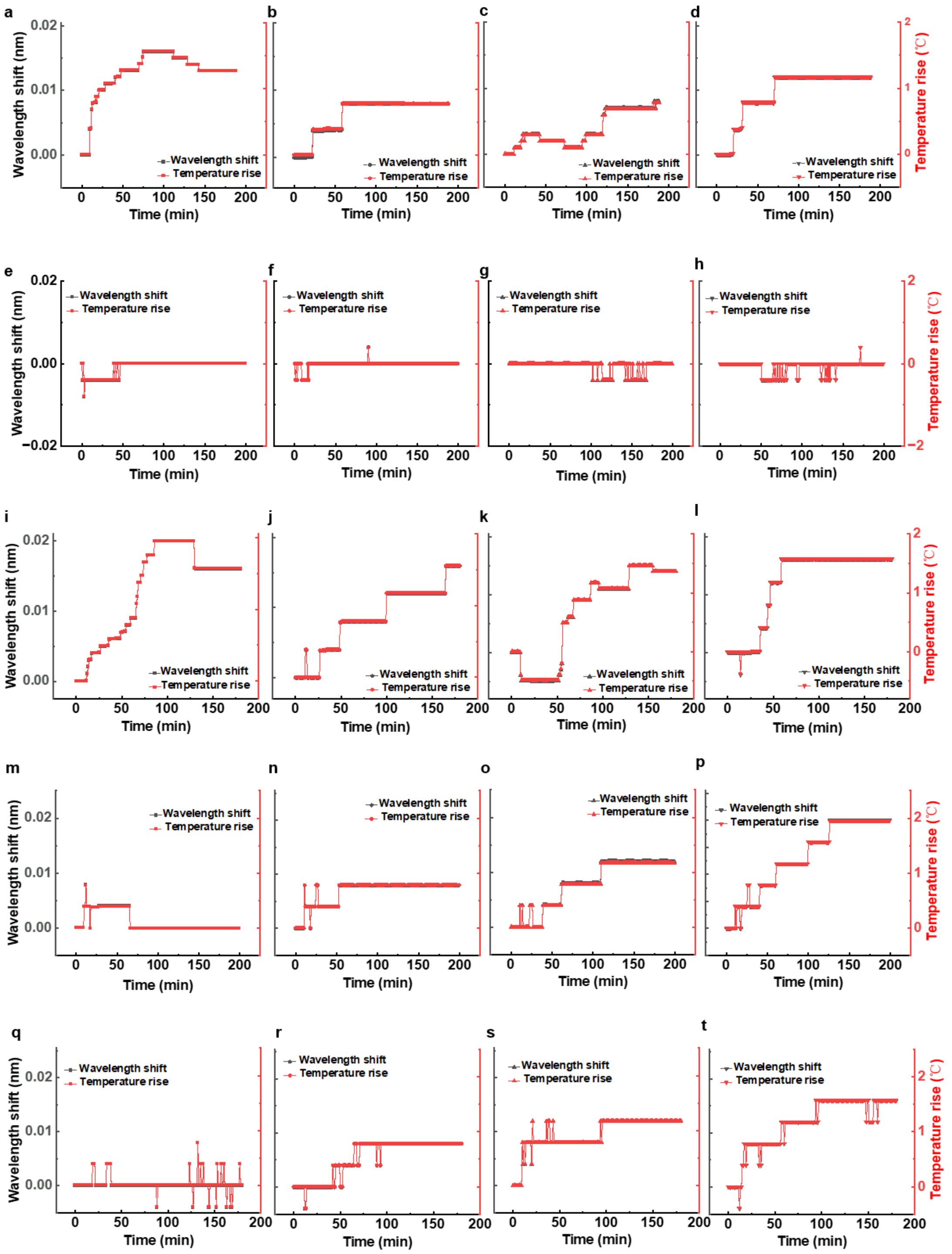

Figure S27. Wavelength shifts of the microFBG versus time (subtraction results) and the corresponding temperature rises in the photoelectrocatalysis process. (**a-d** Under visible light, **e-h** under voltage, **i-l** under visible light + voltage, **o-r** under NIR light, **m-p** under NIR light + voltage; **a, e, i, m, q** Bi_2O_3, **b, f, j, n, r** Bi_2O_3-UCNP0.5, **c, g, k, o, s** Bi_2O_3-UCNP1, and **d, h, l, p, t** Bi_2O_3-UCNP2.)

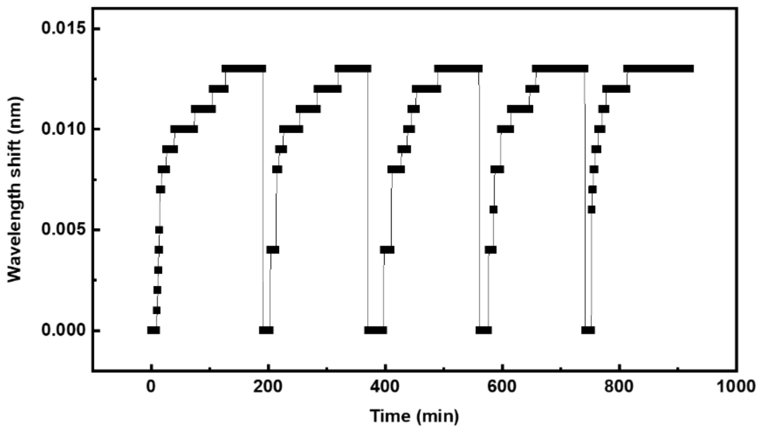

Figure S28. Wavelength shifts of the microFBG versus time in five degradation cycles of tetracycline over Bi_2O_3 (recorded by the lab-around-microfiber sensor).

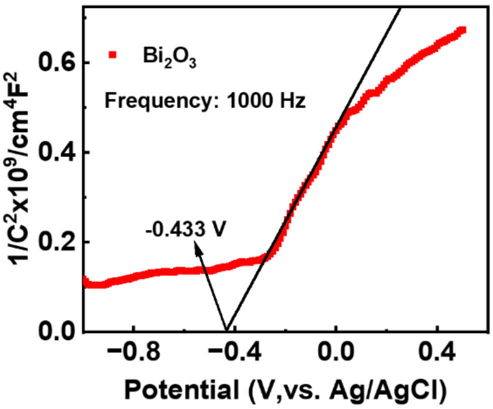

Figure S29. Mott-Schottky plots obtained for the films electrodes prepared with Bi_2O_3 in 0.5 M Na_2SO_4, and the ac amplitude was 10 mV, the frequency was 1000 Hz.

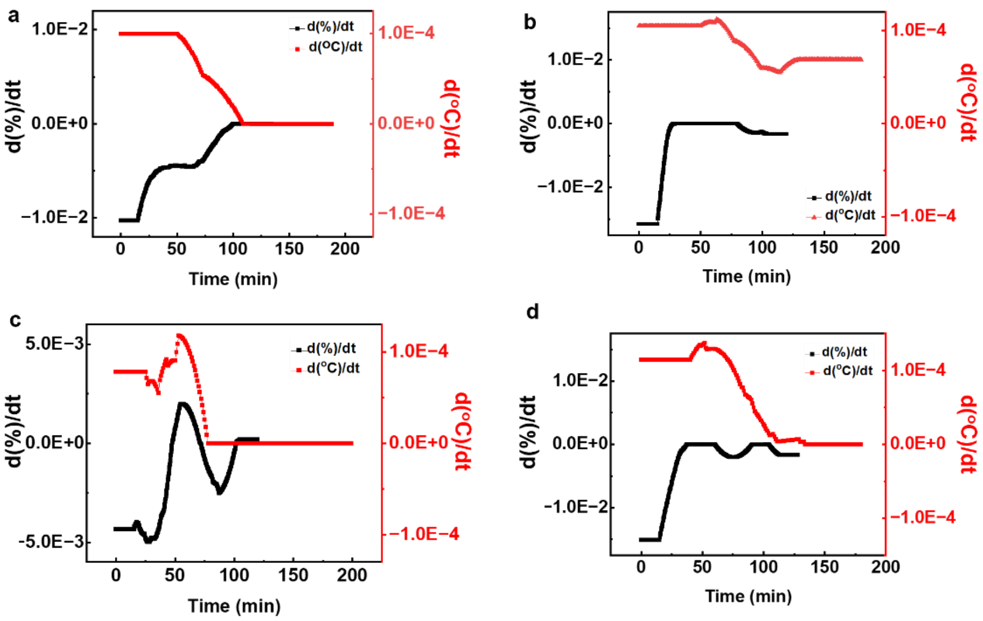

Figure S30. Relationship between spectral derivatives of the pollutant degradation process (($|\lambda_0|$-|△λ|)/$|\lambda_0|$ over time) and derivatives of the temperature changes. Catalyst: Bi_2O_3-UCNP0.5. (a) Under visible light, (b) under visible light + voltage, (c) under NIR light, (d) under NIR light + voltage.
